# Human Induced Pluripotent Stem Cell-Derived 3D-Neurospheres Are Suitable for Neurotoxicity Screening

**DOI:** 10.3390/cells9051122

**Published:** 2020-05-01

**Authors:** Julianna Kobolak, Annamaria Teglasi, Tamas Bellak, Zofia Janstova, Kinga Molnar, Melinda Zana, Istvan Bock, Lajos Laszlo, Andras Dinnyes

**Affiliations:** 1 BioTalentum Ltd., H-2100 Gödöllő, Hungary; julianna.kobolak@biotalentum.hu (J.K.); annamaria.teglasi@biotalentum.hu (A.T.); tamas.bellak@biotalentum.hu (T.B.); Zofia.Janstova@biotalentum.hu (Z.J.); melinda.zana@biotalentum.hu (M.Z.); biotalentum.bock@yahoo.com (I.B.); 2 Department of Anatomy, Cell and Developmental Biology, Eötvös Loránd University, H-1117 Budapest, Hungary; kinga.molnar@ttk.elte.hu (K.M.); laszlo@elte.hu (L.L.); 3 Molecular Animal Biotechnology Laboratory, Szent István University, H-2101 Gödöllő, Hungary; 4 Translational Biomedicine Institute, University of Szeged, H-6720 Szeged, Hungary

**Keywords:** induced pluripotent stem cells, neurospheres, 3D culture, neurite outgrowth, neurotoxicity

## Abstract

We present a hiPSC-based 3D in vitro system suitable to test neurotoxicity (NT). Human iPSCs-derived 3D neurospheres grown in 96-well plate format were characterized timewise for 6-weeks. Changes in complexity and homogeneity were followed by immunocytochemistry and transmission electron microscopy. Transcriptional activity of major developmental, structural, and cell-type-specific markers was investigated at weekly intervals to present the differentiation of neurons, astrocytes, and oligodendrocytes. Neurospheres were exposed to different well-known toxicants with or without neurotoxic effect (e.g., paraquat, acrylamide, or ibuprofen) and examined at various stages of the differentiation with an ATP-based cell viability assay optimized for 3D-tissues. Concentration responses were investigated after acute (72 h) exposure. Moreover, the compound-specific effect of rotenone was investigated by a panel of ER-stress assay, TUNEL assay, immunocytochemistry, electron microscopy, and in 3D-spheroid based neurite outgrowth assay. The acute exposure to different classes of toxicants revealed distinct susceptibility profiles in a differentiation stage-dependent manner, indicating that hiPSC-based 3D in vitro neurosphere models could be used effectively to evaluate NT, and can be developed further to detect developmental neurotoxicity (DNT) and thus replace or complement the use of animal models in various basic research and pharmaceutical applications.

## 1. Introduction

Environmental stressors, such as chemicals or the medical drugs could have a toxic effect on humans which may occur at any stage of their life, during fetal development, childhood, or adult life. The toxic effects of environmental agents coupled with inherited susceptibility of individuals make the toxicology prediction difficult. Conventional animal-based toxicity and safety tests have high costs, use large numbers of animals (mainly rats) and in many cases, they do not provide clearly translatable results for humans [1,2,3]. Consequently, in line with legislation, there is an increasing need to develop alternative testing methods which could handle thousands of drugs or chemicals with affordable time and cost and with human-relevant neurotoxicology (NT) outcome [4,5,6,7]. Development of new approach methods (NAM), would be important both for NT and developmental neurotoxicology (DNT) tests, providing data on the effect of chemicals and the potential adverse outcomes (AOs) [6,8,9,10,11,12,13].

An increasing number of studies use primary cell cultures and recently, pluripotent stem cells (PSCs) to create in vitro systems for NT and DNT screenings. It is widely accepted concept that 3D cell cultures can mimic better the original tissue environment including tissue-specific architecture, mechanical and biochemical features, cell-to-cell communication and signaling, and differentiation capability, while 2D cell culture systems are less complex and more artificial in this sense [14,15,16,17], although the existing assays provide meaningful readouts for specific neurodevelopmental processes [6,8,11]. Only one in vitro assay will not be able to cover the complexity of the in vivo development, therefore a battery of assays and fit-for-purpose applications should be used to cover the relevant processes [10,18]. Human induced pluripotent stem cell-derived (hiPSC) neurospheres, grown on 3D scaffolds or self-forming, can establish cell–cell interactions and model certain neurodevelopmental processes, therefore, might be used as an in vitro screening platform not only for NT but in DNT studies or drug development [19,20,21].

Neural stem cells (NSCs) are considered as a multipotent and self-renewing pool of cells in the mammalian central nervous system (CNS), occurring in vivo in the developing embryonic neural tissue as early as the neural tube formation happens. These cells have the capacity to differentiate into all neuronal cell types, as well as into glial cells, therefore they are able to emulate some fetal neurodevelopmental processes [22,23]. Moreover, NSCs can be differentiated in vitro from pluripotent stem cells (PSCs) thus providing an attractive and almost unlimited in vitro tool for toxicology studies including drug development [24,25].

Despite the developments mentioned above, still, a limited number of human iPSC derived 3D neuronal culture-based studies were published focusing on the development of NT or DNT models [21,26,27,28,29,30,31]. A limiting factor for the further development of such test methods is the lack of high-throughput screening (HTS) read-outs on 3D cell cultures [16], despite the developments using high-content image analysis (HCA) [27].

Here, we present a reliable model system where hiPSC-derived NSCs are differentiated towards subtypes of neurons, astrocytes, and oligodendrocytes, forming free-floating 3D neurospheres in 96-well plate format. Over 6 weeks the various differentiation stages are characterized, and three selected stages are exposed with different compounds to investigate their cytotoxic effect. The generated neuronal spheroids used for NT measurements at different time-points resembling various differentiation stages, therefore, provide an excellent platform for further DNT test system developments.

## 2. Methods

### 2.1. Chemicals and Plasticware

The chemicals were purchased from Sigma-Aldrich (St Louis, MO, USA) and all cell culture reagents and plasticware from Thermo Fisher Scientific Inc. (Waltham, MA, USA), unless otherwise specified.

### 2.2. Human iPSC Culture

Human iPSC line (Ctrl-2), derived from a healthy mid-age Caucasian female donor peripheral blood mononuclear cells (PBMCs), established and characterized earlier [32,33], was used in this study. Cells were cultured on BD Matrigel™ matrix (BD Biosciences, Franklin Lakes, NJ, USA) with mTeSR™1 medium (Stem Cell Technologies, Vancouver, Canada), using Gentle Cell Dissociation Reagent for passages, according to the manufacturer’s instruction. Representative hiPSC colony morphology, pluripotency staining for POU5F1, NANOG, SSEA4, and TRA1-60 and karyotype analysis are presented in Figure S1. For mycoplasma screening, the Venor^®^GeM-Advance (Minerva Biolabs) Mycoplasma Detection Kit was used according to the manufacturer’s protocol in every fifth passage during maintenance and before freezing. Cells were cultured at 37 °C in a humidified atmosphere containing 5% CO_2_. In the current study cultures from passage 16 and 17 were used for differentiation.

### 2.3. Neuronal Differentiation and Maintenance

The Ctrl-2 hiPSC line was differentiated to neural stem cells (NSCs) by dual SMAD inhibition procedure [34], following the detailed protocol of Shi et al. [35]. Briefly, when hiPSC cultures reached 90% confluence, the culture was passaged onto poly-l-ornithine and laminin (POL/L; 0.002%/1 µg/cm^2^) coated plates, using conventional iPSC passage (as detailed above). On the next day media was changed to neural induction medium (NIM; DMEM/F12: Neurobasal medium, supplemented with 1× N2, 2× B27, 2 mM glutamine, 1× non-essential amino acid (NEAA), 100 µM β-mercaptoethanol, 5 µg/mL insulin) supplemented with 10 µM SB431542, 500 ng/mL Noggin (R&D Systems, Inc., Minneapolis, MN, USA) and 5 ng/mL basic fibroblast growth factor (bFGF) to induce the neuroectodermal lineage. Neuronal induction media was applied until day 10 of the differentiation. The forming neural rosette-like structures were manually picked under a stereomicroscope (Olympus SZX2; Olympus Ltd. Tokyo, Japan) and re-plated onto POL/L (POL/L; 0.002%/1 µg/cm^2^) plates. NSCs were expanded in neural maintenance medium (NMM; DMEM-F12: Neurobasal medium, supplemented with 1× N2, and 2× B27, 2 mM glutamine, 1× NEAA), supplemented with 10 ng/mL bFGF and 10 ng/mL Epidermal growth factor (EGF) and maintained in a monolayer on dishes coated with POL/L (0.002%/1 µg/cm^2^). When reached 100% confluence, cells were passaged using Accutase (Sigma) and seeded as single cells (50,000 cells/cm^2^) for further expansion on POL/L (0.002%/1 µg/cm^2^) coated dishes. After 4 passages NSCs were frozen in 1 mL freezing medium (90% Fetal Bovine Serum, FBS; heat-inactivated, Thermo Fisher Sci.; Cat N.: 10500-064, LOT: 08F1180K; 10% DMSO; 2 million cells/vial), using Accutase passage. Before freezing the NSCs, a mycoplasma test was performed (see above), repeated every fifth passage during maintenance.

### 2.4. 3D Neurosphere Culture

After thawing, NSCs (generated as detailed above) were cultured on POL/L (0.002%/1 µg/cm^2^) coated plates (50,000 cells/cm^2^). When reached 100% confluence NSCs were propagated with Accutase treatment and single-cell suspension was plated onto a low-adherent 96-well plate with 10,000 cells/well in NMM medium. Neurospheres were formed within 48 h after plating and NMM half of the media was changed in every 3 days until analyzing the samples. Samples were collected at Day 0 (D0), Day 2 (D2), and weekly intervals from the end of the 1st week (D7) until the end of the 6th week (D14, D21, D28, D35, and D42).

### 2.5. Cryosectioning and Immunocytochemistry (ICC) Staining

3D neurospheres (untreated, vehicle or compound-treated) were fixed with 4% paraformaldehyde (PFA) in 0.1 mol/L phosphate buffer for 1 h at RT and washed 3 times with PBS. The fixed samples were cryoprotected in 30% sucrose in PBS containing 0.01% sodium azide at 4 °C until embedding in Shandon Cryomatrix gel (Thermo Fischer Scientific). The 16 µm parallel sections were made using cryostat (Leica CM 1850 Cryostat, Leica GmbH), mounted to Superfrost™ Ultra Plus Adhesion Slides (Thermo Fisher Scientific) and stored at −20 °C until use. After 10 min air-drying, the sections were blocked for 1 h at RT with blocking solution (3% BSA in PBS), supplemented with 0.2% TritonX-100. The sections were then incubated with primary antibodies (Table S1) overnight at 4 °C. Next day, sections were washed in PBS 3 times, and isotype-specific secondary antibodies (Table S1) were diluted in blocking buffer and applied for 1 h at RT. The sections were washed 3 times with PBS and covered using Vectashield^®^ mounting medium containing DAPI (1.5 µg/mL; Vector Laboratories), that labelled the nuclei of the cells (at least 1 h at RT). Negative controls for the secondary antibodies were performed by omitting the primary antibodies. Immunoreactive sections were analyzed using a BX-41 epifluorescent microscope (objectives: 20× 0.50 NA; 40× 0.75 NA; Olympus) equipped with a DP-74 digital camera and its CellSens software (V1.18; Olympus). For confocal imaging, Olympus Fv10i-W compact confocal microscope system (objective: 60× 1.35 NA; Olympus) with Fv10i software (V2.1; Olympus) was applied. All images were further processed using the GNU Image Manipulation Program (GIMP 2.10.0) and NIH ImageJ analysis software (imagej.nih.gov/ij).

Quantification of the immunocytochemistry data was performed using ImageJ software according to Tieng et al. [36]. Briefly, images were taken by confocal microscopy at 120× magnification. The numbers of Ki-67-, NESTIN-, GFAP-, AQP4-, TUBB3-, NF200kD-, MBP-, VAMP2-, and MAP2-immunoreactive pixels were measured in 5 neurosphere (highest diameter middle sections, 5 randomly selected fields/slide) at every time points. Data was normalized with DAPI positive nuclei number. Data were expressed as a percentage of marker/DAPI ratio ± SEM (*p* < 0.05).

### 2.6. Apoptosis Assay

Embedding and cryosectioning of 3D samples were performed as above. To detect apoptotic activity, the DeadEnd™ Colorimetric TUNEL System (Promega) was used on the middle cryosections (highest diameter) of the spheroids, following the instructions of the manufacturer. In brief, apoptosis was detected by immersing the slides in PBS for 5 min (at RT), adding 20 μg/mL Proteinase K solution and incubating for 10–30 min (at RT). After 5–10 min treatment in Equilibration buffer, recombinant terminal deoxynucleotidyl transferase (rTdT) was added to the reaction mixture. Next, the sections were incubated for 60 min at 37 °C inside of a humidified chamber to allow the end-labelling reaction to occur. The reaction was terminated by immersing the slides in saline-sodium citrate for 15 min (RT). Endogenous peroxidases were blocked by immersing the slides in 0.3% hydrogen peroxide in PBS for 3–5 min (RT). Streptavidin-HRP was added to slides, incubated for 30 min (RT), stained with diaminobenzidine (DAB) solution for 5 min until a light brown background appeared. For hematoxylin–eosin (HE) staining Mayer’s Hematoxylin solution was used for 3 min. Sections were rinsed with tap water and placed into distilled water for 30 s, then into 96% alcohol for 30 s. One percent Eosin solution in distilled water was used for 3 min. Stained sections were dehydrated through alcohols, clear in xylene and mount in DPX. Microphotographs were made with DP-74 digital camera (Olympus) using a light microscope (BX-41, objectives: 20× 0.50 NA; 40× 0.75 NA; Olympus) and CellSens software (V1.18; Olympus). For counting the apoptotic and total (Hematoxylin-stained) number of cells, NIH ImageJ analysis software was used. Five Ctrl and five ROT-treated spheroids were randomly selected, and middle sections were analyzed from each differentiation stage (D21, D28, and D42) samples in three experiments (*n* = 3).

### 2.7. Transmission Electron Microscopy (TEM)

Neurospheres (untreated, vehicle or compound-treated) were fixed at different differentiation stages in a fixative solution containing 3.2% PFA, 0.2% glutaraldehyde, 1% sucrose, 40 mM CaCl_2_ in 0.1 M cacodylate buffer (pH 7.4) for 12 h at 4 °C. Samples for ultrastructural analysis were embedded in 1.5% agar (dissolved in dH_2_O), post-fixed in 1% ferrocyanide-reduced osmium tetroxide [37], then dehydrated using graded series of ethanol, finally embedded in Spurr low viscosity epoxy resin medium. Ultrathin sections were collected from the middle region of the spheroids (highest diameter) on copper slot grids coated with formwar (Agar Sci., Essex, UK) and counterstained with uranyl acetate and Reynolds’s lead citrate. Sections were examined with a JEOL JEM 1011 transmission electron microscope (JEOL Ltd., Tokyo, Japan) equipped with a Morada 11-megapixel camera using iTEM software (Olympus).

### 2.8. RT-qPCR Analysis

For each sample, 12 spheroids were pooled, and 3 biological replicates were performed (*n* = 3). Total RNA was isolated using the RNeasy Plus Mini Kit (Qiagen, Hilden, Germany). For the reverse transcription, 600 ng of the isolated RNA was used applying the Maxima First Strand cDNA Synthesis Kit for RT-qPCR with dsDNase (Thermo Fisher Scientific) according to the manufacturer’s instructions.

Gene-specific primers were designed using the Primer3 software [38], specified with mFOLD software [39] and Primer-BLAST software [40]. Primers were optimized using two-fold serial dilution standard curves (Table S2). As a reference, gene GAPDH was used (Table S2). Each real-time PCR reaction contained 5 ng RNA-equivalent cDNA template, 400 nM of each primer and 50% SYBR Green JumpStart Taq ReadyMix (Sigma Aldrich) in a total volume of 15 μL. PCR reactions were set up using QIAgility liquid handling robot and performed on a Rotor-Gene Q cycler (Qiagen). The cycling parameters were as follows: 94 °C for 3 min initial denaturation followed by 40 cycles of 95 °C for 5 s, 60 °C for 15 s, and 72 °C for 30 s. Melting curve analysis and agarose gel electrophoresis confirmed the specificity of the primers and the absence of gDNA contamination. Data of three replicates were analyzed for each gene, using the ddCT method [41].

### 2.9. XBP1-Assay of Endoplasmic Reticulum Stress

Upon accumulation of unfolded proteins in the endoplasmic reticulum (ER), a 26-nucleotide fragment from the X-box binding protein 1 mRNA (XBP1(U)) is removed with a special splicing mechanism [42]. This shorter mRNA (XBP1(S)) is a frequently used marker of ER-stress. To study the expression of XBP1(S), previously described primers [43] and our own primers were used [44] (Table S2). As a positive control, cells were treated with 5 µM and 10 µM Tunicamycin to induce ER-stress. RT-PCR reactions were performed and analyzed as above, using Phusion Hot Start II High-Fidelity DNA Polymerase and 20 ng of the cDNA samples.

### 2.10. Toxicity Treatments and ATP Viability Assay

Eleven well-known compounds were tested in 7 different concentrations to generate concentration-response curves. The tested compounds with the used concentrations are detailed in Table S3. The highest dosage was determined based on the solubility of the compounds, and care was taken not to reach above 0.1% DMSO levels after diluting the compound in the culture media. In each assay plate, 4 technical replicates were applied from each sample and 3 biological replicate assays were run (*n* = 3). The 3D cell cultures were exposed to the toxicants for 72 h (exposure schemes are detailed in Figure 5A or Figure 6A or Figure 7A). Vehicle control was used as a regular medium supplemented with 0.1% DMSO.

ATP viability assay was performed with CellTiter-Glo^®^ 3D Cell Viability Assay, according to the manufacturer’s protocol (Promega). Neurospheres were lysed with 100 μL CellTiter-Glo^®^ 3D Reagent for 60 min at RT. Luminescence signal was recorded with a Thermo VarioScan Flash (Thermo Fisher Scientific) plate reader.

### 2.11. Diametric and Total Protein Determination of the Spheroids

3D spheroids grown in a 96-well plate were captured using the 4x objective (0.1 NA) of Olympus IX71 microscope and DP21 camera (Olympus). The images were analyzed by measuring the diameters of the spheroids using the Olympus CellSens Dimension software (V1.11). Each value represents the average of 3 experiments (*n* = 3) in each 96 spheroids were measured. The 3D spheroids were lysed individually with RIPA Lysis and Extraction Buffer supplemented with Halt™ Protease and Phosphatase Inhibitor Cocktail and Pierce™ Universal Nuclease for Cell Lysis, sonicated and the total protein concentration determined using a Pierce BCA Protein Assay Kit according to the manufacturer’s instructions. Due to the small spheroid size of the D2, D7, and D14 samples, three spheroids were pooled, then the individual values were calculated accordingly. In total, three experiments were performed (*n* = 3), in which 24 spheroids were analyzed for each timepoint.

### 2.12. Neurite Outgrowth Assay

For neurite outgrowth measurement the method of Harris et al. was modified [45]. 3D spheroids were treated with different concentrations (vehicle, 0.1 µM, 0.5 µM, and 0.75 µM) of Rotenone (ROT) at D21 differentiation stage using the 72 h exposure scheme, similarly to the previous concentration-response experiments. After the treatment, the spheroids were plated on Matrigel-coated plates in NMM medium without compound, and 24 h later fixed with 4% PFA and immunostained against TUBB3, as described in detail under the immunostaining paragraph. First, the number of neurites was counted for each sample. Then, the “edge” of the spheroids was determined by drawing an “edge line” and every neurite length was measured from this “edge line” to the tip of each neurite. Total neurite length was calculated for each spheroid using the ImageJ software’s Neurite Tracer plugin. In each experiment eight spheroids were treated in each experimental group (5 groups) and three independent experiments were performed (*n* = 3).

### 2.13. Statistical Analysis

All results were analyzed using Prism 5 (GraphPad Software, La Jolla, CA, USA) and handled in Microsoft Office 2010 (Microsoft, Redmond, WA, USA) software. For normalization of the concentration response, the average values of positive control were used as 0% and the average of vehicle control served as 100%. Four parameter curve fitting methods were used to determine EC10 and EC50 values, where the data on the graphs represent the average of three biological replicates. The “n” value corresponds to the number of biological replicates for each tested concentration. Analysis of data is presented as the mean ± SEM. Significance of data was determined with paired T test for RT-qPCR (* *p* < 0.01) and with One-way ANOVA for concentration response (* *p* < 0.05; ** *p* < 0.01).

## 3. Results

### 3.1. Three-Dimensional Spheroid Differentiation of iPSCs-Derived NSCs Revealed Complex Neuronal Cultures

Our first aim was to characterize the timewise differentiation of 3D neuronal spheroids free-floating in suspension culture, originating from hiPSCs. The neuronal differentiation capacity of the starting neural stem cell (NSCs) population, derived by the dual-SMAD inhibition protocol, was comprehensively characterized by us previously [32,33]. These cells expressed the major NPC markers (NESTIN, SOX1 (SRY-Box 1), PAX6 (Paired Box 6) investigated on protein level with ICC (see details in Figure S2). When the NPCs were terminally differentiated in a 2D culture system over 5 weeks, cortical neurons and glial cells were differentiated and formed a neuronal network, as we published recently [32]. In the present study differentiation in a 3D culture system was investigated in the course of 6 weeks (42 days).

**Figure 1:**
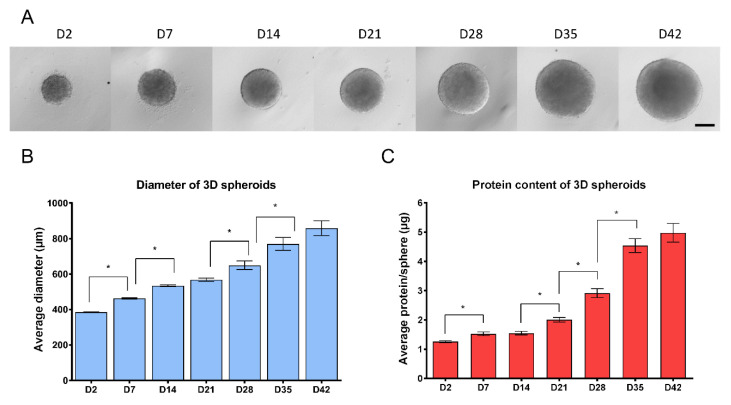
Growth properties of 3D neurospheres during the 6 weeks of differentiation (**A**) Representative microscopic view of 3D spheroids, investigated at weekly intervals (4×, scale bar: 200 µm). (**B**) The average diameter of 3D spheroids in µm measured by CellSens Dimension software (Olympus) (*n* = 3, in each experiment 96 spheroids were compared weekly trough 6-weeks). (**C**) Average total protein content of spheroids determined by Pierce BCA Protein Assay. Note that D2, D7 and D14 samples were measured by pooling three spheroids and individual values were calculated, while in all other timepoints spheroids were measured individually (*n* = 3, in each stage 24 spheroids were measured). ±SEM values are presented on graphs. (* *p* < 0.05).

In suspension culture upon withdrawing the two mitogens EGF and bFGF, NSCs formed compact 3D spheroids, the so-called neurospheres within 48 h (Figure 1A). Growth properties of the structures were investigated by measuring the diameter of the individual spheres and their total protein content. Results showed a continuous growth of the spheres during the 6 weeks culture period, in terms of diametric growth and protein content (Figure 1). While the diameter increased linearly, the protein content increased more dynamically after week 4, resembling the cellular and structural changes during differentiation (Figure 1B,C).

**Figure 2:**
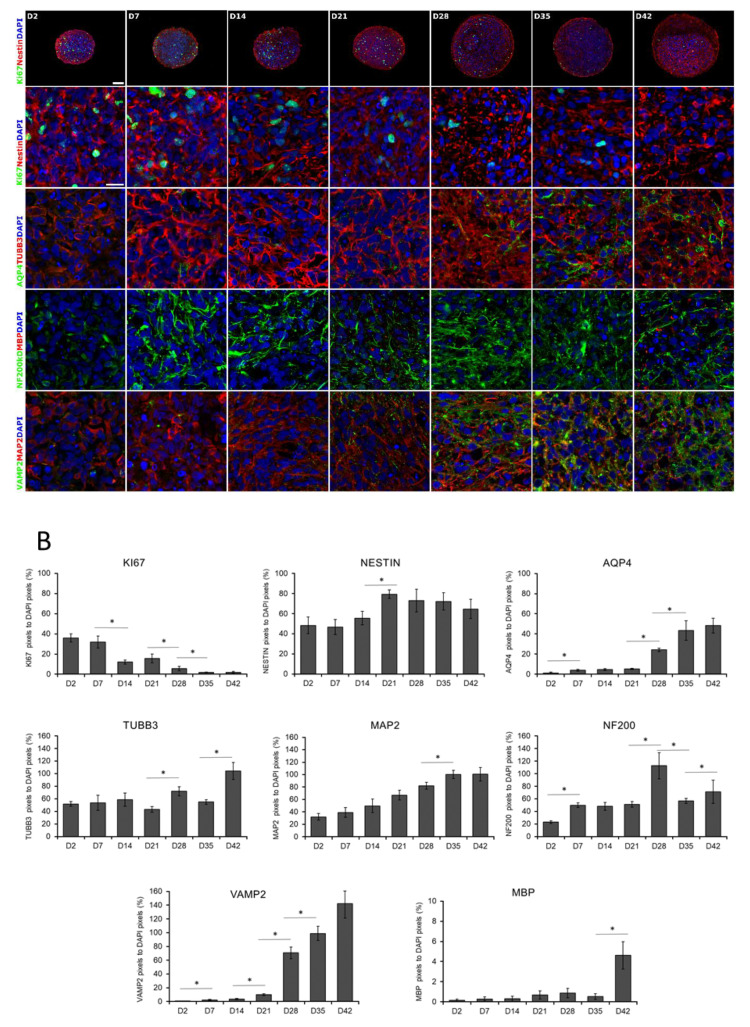
Immunocytochemical analysis of 3D spheroids. (**A**) Spheroids were fixed and cryosectioned then immunostained at weekly intervals from D2 until D42 stage. The first line represents the overview of the cryosectioned spheroids, while the rest of the panel shows higher magnifications. Relevant markers of proliferation (KI67), neural stem cells (NESTIN), neuronal differentiation (TUBB3 and MAP2), an intermediate filament of dendrites and axons (NF200), synaptic vesicles of neurons (VAMP2), astrocyte (AQP4), and oligodendrocyte (MBP) specific proteins were stained. Protein name IDs are indicated with colors, representing the color of the fluorophore used (e.g., green as Alexa 488; red as Alexa 594) Nuclei were counterstained with DAPI (in blue). Scale bar: 100 µm (first line only) and 25 µm. (**B**) Quantitative analysis of the immunostainings on confocal images. The numbers of Ki-67, NESTIN, AQP4, TUBB3, NF200kD, MBP, VAMP2, and MAP2 immunoreactive pixels were measured in 5 neurospheres (middle sections, 5 randomly selected fields/slide) at every time points. Data was normalized with DAPI positive nuclei number. Data were expressed as percentage of marker/DAPI ratio ± SEM (* *p* < 0.05).

**Figure 3:**
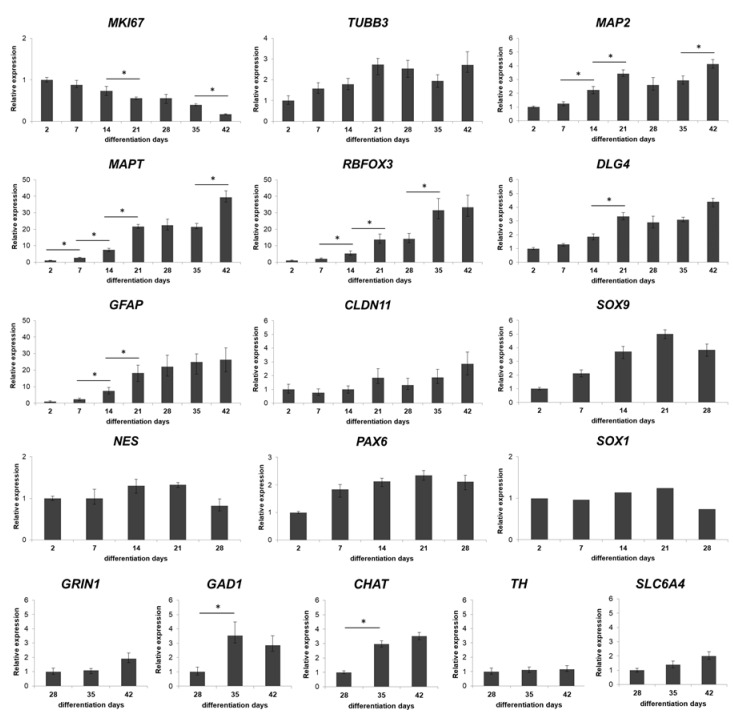
Real-time PCR measurements of relevant markers during the neuronal differentiation of the spheroids. Twelve 3D spheroids were pooled, lysed with RLT-buffer and used in RT-PCR analysis at each timepoints. Where the genes are already expressed in D2 samples, it is referred as 1. Day 2, 7, 14 and 21 values for *GRIN1, CHAT, TH, GAD1,* and *SLC6A4* are not indicated as these genes were not expressed at those time points. Graphs represent normalized relative expression values, analyzed for each gene using the ddCT method [41]. Mean values and ± SEM of three biological replicates (*n* = 3) are presented on graphs, significance are determined by paired T test (∗ *p* < 0.01). Note that y axis’s scales alter according to the different relative expression values.

This observation was in accordance with the expression of the cell proliferation marker Ki-67 (KI67), representing the proportion of dividing cells, monitored by ICC on protein level (Figure 2A,B) or by RT-qPCR on transcript level (Figure 3). The expression of Ki-67 and the number of Ki-67-positive cells were the highest in D2 and D7 samples, which showed a continuous decline over the differentiation period (Figure 2A,B or Figure 3). The forming 3D cell aggregates mainly expressed the early neuronal markers such as *PAX6*, *NESTIN*, and *SOX1*, which expressions decreased with maturation on a time-wise manner (RT-qPCR, Figure 3). Tubulin beta-3 chain (TUBB3) expression appeared very early in the samples, already at D2 as neuronal processes started to grow inside the spheres (ICC, Figure 2A,B). These changes were obvious when investigated at transcript level as well (RT-qPCR, *TUBB3*; Figure 3). It is important to note that no necrotic regions were detected in the center of the spheroids or in other regions, not even the highest diameter of spheroids reached 800 µm in average in D42 samples (Figure 1B or Figure 2 first line). This fact was corroborated in semithin sections where cell density proved to be high and consistent during the whole examined period. Layer organized fibers had become distinguishable on the surface from the 21st day (Figure S3a–g).

A weekly investigation of the gene expression revealed a continuous progress in neuronal differentiation and increase in the level of maturation markers, such as the dendritic marker Microtubule-associated protein 2 (*MAP2*) or Microtubule-associated protein tau (*MAPT*), and RNA Binding Fox-1 Homolog 3 (*RBFOX3*, also known as NeuN) (Figure 3), which were in accordance with the protein data of immunofluorescent staining of the 3D spheroids, MAP2 and Neurofilament 200 kDa (NF200); (ICC, Figure 2A,B).

**Figure 4:**
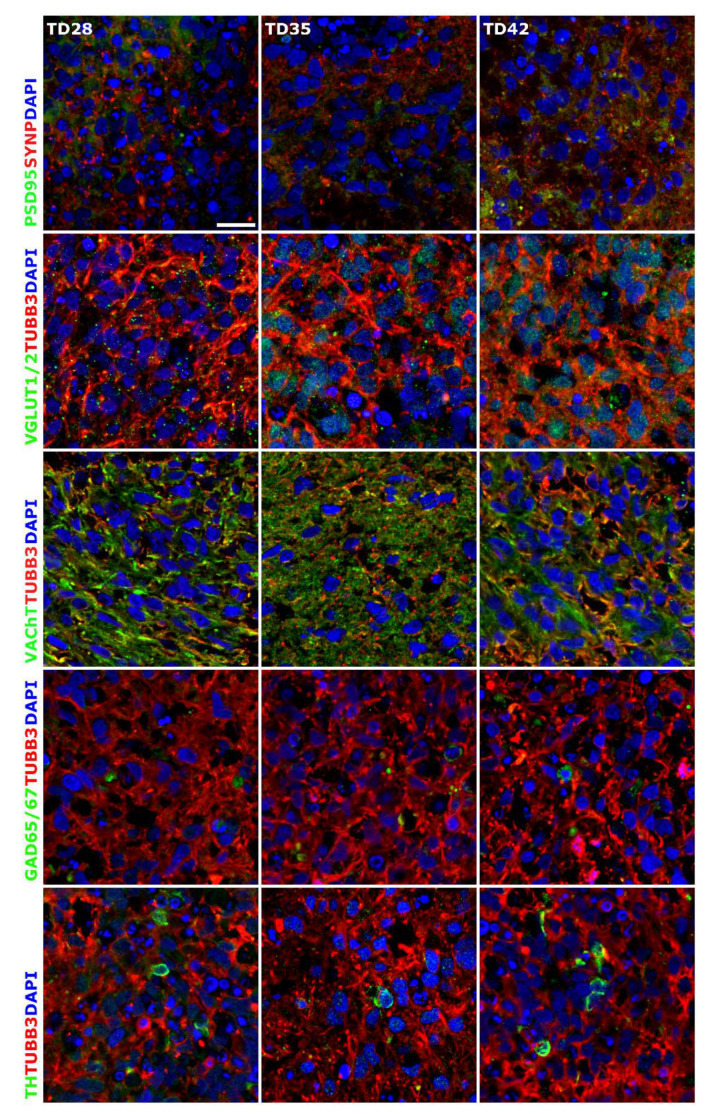
Immunocytochemical detection of neuronal subtypes in 3D neurospheres. Presence of synapses was determined with post-synaptic marker PSD95 and synaptic protein Synaptophysin (SYNP) double staining. Glutamatergic (VGLUT1/2), GABAergic (GAD65/67), cholinergic (VAChT) and dopaminergic (TH) neurons were detected in the developing 3D neurospheres from D28. All samples were stained with TUBB3 (in red) to label the neurites. Protein name IDs are indicated with colors, representing the color of the used fluorophore (e.g., green as Alexa 488; red as Alexa 594). Nuclei were counterstained with DAPI (in blue). Scale bar: 25 µm.

Terminal differentiation resulted in the formation of neuronal networks and synapses, from the stage of D28, post-synaptic marker PSD95 (now DLG4, Discs Large MAGUK Scaffold Protein 4) (Figure 3 and Figure 4), and the major synaptic vesicle protein p38, Synaptophysin (SYNP) and Vesicle-associated membrane protein 2 (VAMP2) (Figure 2 and Figure 4) expression appeared in maturing neurons. Synapse formation was assessed by transmission electron microscopy (TEM) revealing the presence of synaptic connections, presynaptic vesicles and postsynaptic density (Psd). Matured synapses were detected in D42 samples, where docked presynaptic vesicles were determined by identifiable contact point between the vesicle and the presynaptic membrane (Figure S3h,i).

Neuronal subtypes were investigated by the expression of glutamatergic (VGLUT1/2), GABAergic (GAD65/67), cholinergic (VAChT) and dopaminergic (TH) neurons with ICC and qRT-PCR (*GRIN1*: Glutamate Ionotropic Receptor NMDA Type Subunit 1; *GAD1*: Glutamate Decarboxylase 1; *CHAT*: Choline *O*-Acetyltransferase; *TH*: Tyrosine Hydroxylase; *SLC6A4*: Solute Carrier Family 6 Member 4) (Figure 3 and Figure 4). The expression of these markers was detectable mainly from D28 stage and gradually increased until the last investigated time point of the differentiation (D42), in line with the maturation process. It has to be remarked that the spontaneous differentiation of dopaminergic neurons was rare in the cultures, we identified only a few TH positive cells in the cultures (Figure 4).

During the neuronal differentiation, astrocytes started to emerge after week 2, based on the Aquaporin-4 (AQP4) or the Glial fibrillary acidic protein (GFAP) expression both at mRNA and protein level (Figure 2, Figure 3 and Figure S4 ). Oligodendrocyte differentiation followed a similar course, from D21 it was clearly detectable both at transcript (*Claudin 11*, *CLDN11*, formerly known as Oligodendrocyte marker 4; Figure 3) and protein level (Myelin basic protein, MBP; Figure 2A,B) which gradually increased from D35 with the maturation of the cells, however, still remained in a low expression level (Figure 2A,B or Figure 3). Our ICC results clearly show that the expression of mature neuronal and glial markers and neuron-neuron and neuron-glial interactions increased during the differentiation period, thus emulating the development of the human fetal neural tissue.

Overall, results at the transcript level were in accordance with those of the proteins detected by ICC, confirming the effective differentiation of hiPSC-derived NSCs towards the neural lineage. The 3D neurospheres represented a neuronal tissue-like differentiation, containing neurons, astrocytes and oligodendrocytes, what was presented by transcript-, protein-, and ultrastructural level as well. We can conclude that the 3D spheroid system provides a complex neuronal cell culture which can serve as a model of the early neuronal differentiation.

### 3.2. Early 3D Neurospheres as a Neurotoxicity Model

**Figure 5:**
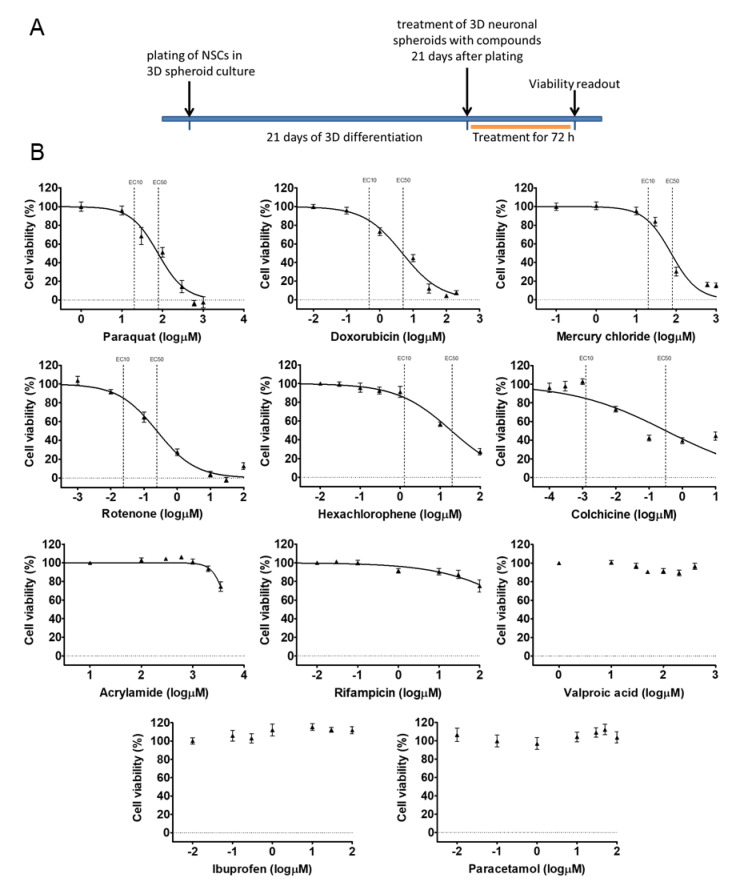
Cell viability measurement on D21 3D neurospheres after 72 h exposure. (**A**) Exposure scheme at D21 stage. (**B**) Concentration response curves of compounds, tested in 7 different concentrations (see concentrations listed in Table S3), representing the cell viability (%) of treated D21 3D neurospheres (*n* = 3). Concentration values are presented in log µM ± SEM. EC10 and EC50 values are presented on graphs where applicable.

Our 3D spheroid-based model was tested in cytotoxicity assay, monitoring the viability of the cells within the neurospheres. Eleven compounds with well-known effects (drugs, pesticides, and chemicals) were selected and applied on the neurospheres in 7 different concentrations (compounds and concentrations are detailed in Table S3) at D21 by acute (72 h) exposure. Concentration-response curves were generated and evaluated. The exposure scheme is presented in Figure 5A.

According to the results, Paraquat (PQ; EC50: 1.89 log µM), Rotenone (ROT; EC50: −0.61 log µM), Mercury(II) chloride (HgCl_2_; EC50: 1.87 log µM) and Doxorubicin (DOX; EC50: 0.67 log µM) caused maximum cell death, Hexachlorophene (HE; EC50: 1.26 log µM) and Colchicine (COL; EC50: −0.49 log µM) had a strong effect on the viability, but did not kill all the cells in the investigated concentration range. Acrylamide (ACR; EC50: 3.5 log µM) and Rifampicin (RIF; EC50: > 2 log µM) also reduced the viability, while Valproic acid (VPA; EC50: > 2.6 log µM) and Paracetamol (PAR; EC50: > 2 log µM) had minimal effect on the viability of 3D neurospheres at D21 stage. Ibuprofen (IBU; EC50: > 2 log µM) as a non-neurotoxic agent was applied as negative control which indeed, did not decrease the viability of D21 3D neurospheres (Figure 5B, Table S4). Overall, the different compounds induced different levels of cytotoxicity in a concentration-dependent manner on the 3D neurosphere cultures.

### 3.3. Different Age of the 3D Neurospheres Represent Distinct Differentiation Stages in the Cytotoxicity Model

**Figure 6:**
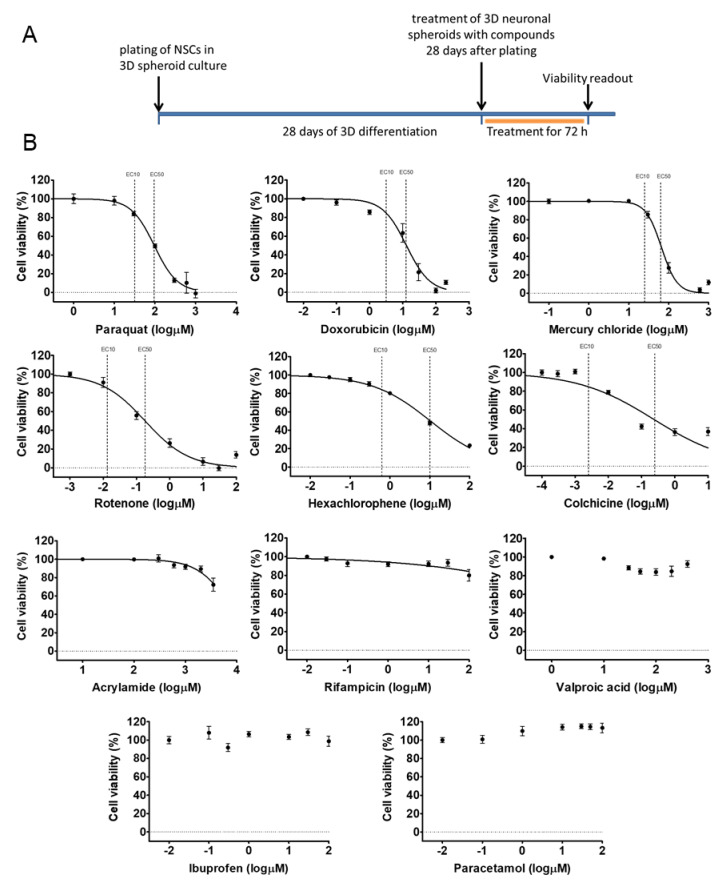
Cell viability measurement on D28 3D neurospheres after 72 h exposure with toxicants. (**A**) exposure scheme at D28 stage. (**B**) Concentration-response curves of compounds, tested in 7 different concentrations (see concentrations listed in Table S3), representing the cell viability (%) of treated D28 3D neurospheres (*n* = 3). Concentration values are presented in log µM ± SEM. EC10 and EC50 values are presented on graphs where applicable.

**Figure 7:**
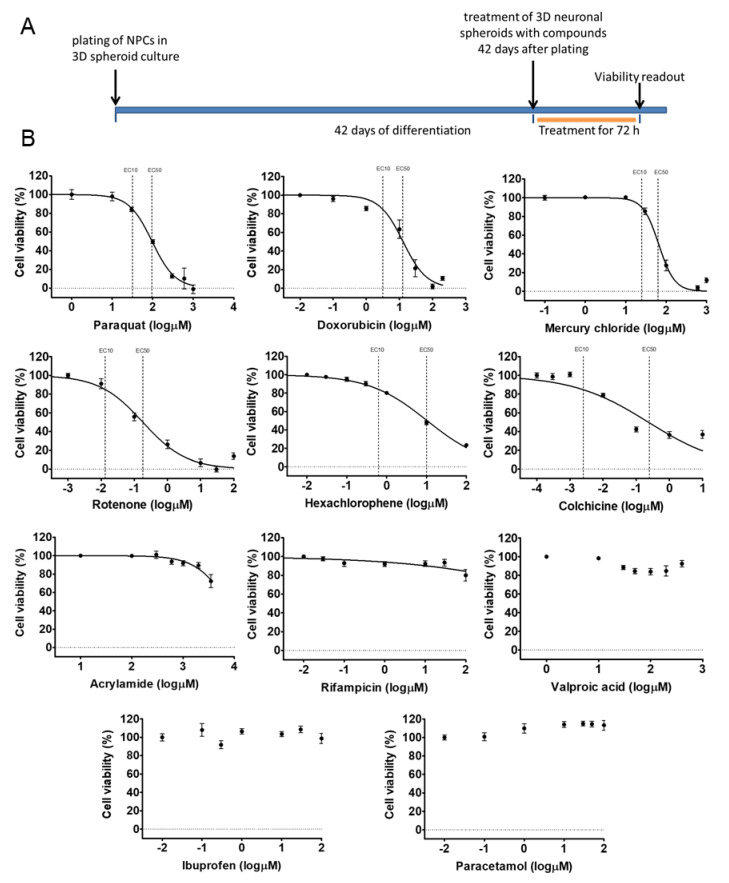
Cell viability measurement on D42 3D neurospheres after 72 h exposure with toxicants. (**A**) exposure scheme at D42 stage. (**B**) Concentration-response curves of compounds, tested in 7 different concentrations (see concentrations listed in Table S3), representing the cell viability (%) of treated D42 3D neurospheres (*n* = 3). Concentration values are presented in log µM ± SEM. EC10 and EC50 values are presented on graphs where applicable.

Next, we investigated the concentration-response of different differentiation stage-derived 3D neurospheres. D28 and D42 samples were analyzed in exposure schemes similar to that of the D21 samples (Figure 6A or Figure 7B) for all the previously tested compounds. As detailed above, in D28 samples differentiated astrocytes expressing GFAP and AQP4 are present (Figure 2 and Figure S4), axonal outgrowth is prevalent, and neuronal subtype-specific proteins start to appear (Figure 2, Figure 3 and Figure 4). The D42 samples represent a more mature cell culture where synapsis of neurons was formed with established subtypes, mature astrocytes and a few oligodendrocytes are already present (Figure 2, Figure 4, Figures S3 and S4).

**Figure 8:**
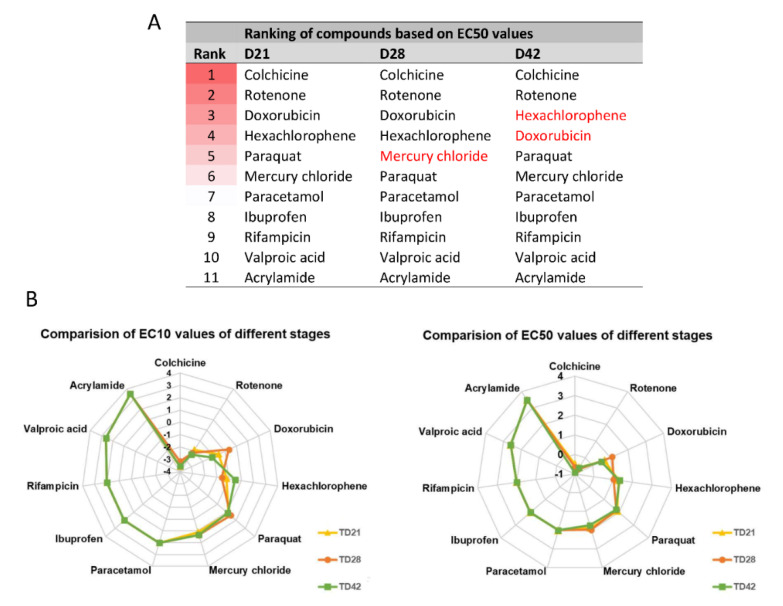
Comparison of the toxic effect of compounds. (**A**) Ranking of compounds at different differentiation stages based on EC50 values. Darker background color represents higher toxicity, while white color represents non-toxic compounds. (**B**) Radar chart showing the comparison of compounds based on the order of EC10 or EC50 values, at various differentiation stages (yellow line with rectangular: D21; orange line with circle: D28; green line with square: D42) (see also EC values in Table S4). Concentration values are presented in log µM.

The experiments showed that both D28 and D42 stages are suitable for the viability assays without modifications in the culture system, using a 96-well plate format-based analysis. The EC50 and EC10 values for D28 and D42 samples were determined for each compound, as described above for D21 samples (Figure 6B or Figure 7B, Table S4). Some compounds (Figure 5, Figure 6 and Figure 7, Table S4) showed less toxic effect on more matured samples (e.g., ACR: EC50^D21^ = 4.1 µM vs. EC50^D42^ = 3.6 µM) while it was the opposite action for other compounds (e.g., HgCl_2_: EC50^D21^ = 1.7 µM vs. EC50^D42^ = 1.9 µM). Based on the data obtained, a concentration-related ranking of the toxicant could be generated for each differentiation stage (Figure 8, Table S4).

In conclusion, we can state that a compound-specific concentration-response was detected in all stages. At the same time, a differentiation stage-relevant difference was observed, suggesting that the in vitro system can mimic the differential responses of the developing fetal human neuronal tissue, caused by distinct toxicants.

### 3.4. Compound-Specific Cellular Events Can be Detected in the 3D Neurospheres

**Figure 9:**
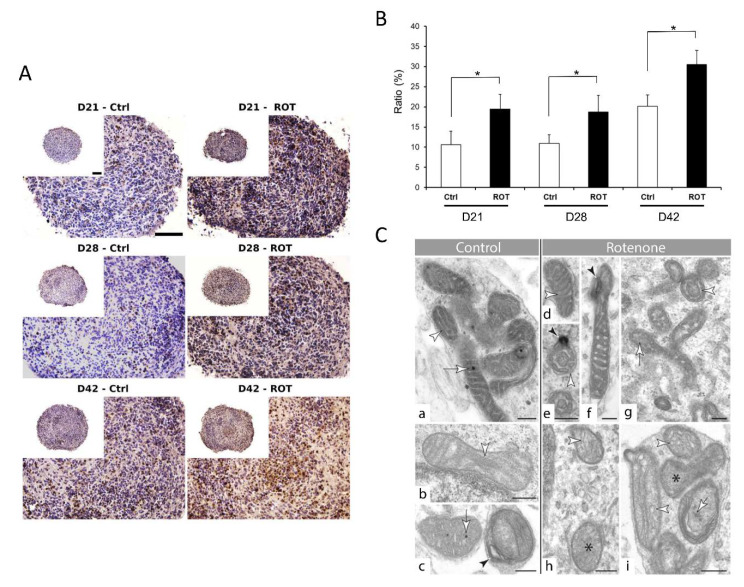
Effect of Rotenone (ROT) exposure on 3D neurospheres at three differentiation stages. (**A**) 3D neurospheres were treated with 0.5 µM ROT concentration for 72 h at three different maturation timepoints (D21, D28, and D42), fixed, sectioned and analyzed to detect the cellular effect of ROT by TUNEL assay (DeadEnd™ Colorimetric TUNEL System, Promega), compared to the vehicle (0.1% DMSO) treated control (scale bar: 100 µm). (**B**) ROT treatment revealed in average a 15% increase in the apoptotic cell number compared to the control in each stage (* *p* < 0.05). Average values are presented on graphs (*n* = 3). (**C**) Ultrastructure of mitochondria in control (panel a, b, c) and ROT treated (d–i) neurons in the 3D spheroids. See the alteration of the internal membrane (cristae) morphology (white arrowheads) in ROT treated cells (d, e, h, i). Black star: unidentifiable cristae morphology in lighter mitochondria (h, i); black arrowhead: membrane swirl in darker organelles (e, f); white arrows: matrix with and without matrix granules (control cells: a, c; treated cells: g, i) Note the density difference between these granules in control (c) and ROT treated mitochondria (panel i) (D21: a, d, f, h; D28: i; D42: b, c, g) (scale bar: 250 nm).

ATP-based viability assay can detect cell death but is unsuitable for detecting other specific cellular events. Due to the lack of validated tests for 3D tissues, we decided to investigate the effect of a toxic compound at a subcellular level. We have chosen rotenone (ROT), a well-studied neurotoxic compound, known to interfere with the electron transport chain in mitochondria, to investigate the ultrastructure of the cells and especially mitochondria, followed by TEM. In parallel, a TUNEL assay was also applied to detect cell death. Based on the previously determined concentration-response curves and EC50 values, we treated the 3D neurospheres with 0.5 µM ROT for 72 h and analyzed the samples in the 3 differentiation stages. The data showed that ROT significantly increased the cell death in the spheroids (Figure 9A or Figure S6), compared to the vehicle-treated controls (Figure 9B), in accordance with the results of the ATP measurement (see Figure 5B or Figure 6B). Moreover, TEM revealed a change in the ultrastructure of the mitochondrial inner membrane, the cristae: Both shape and complexity were changed in the ROT treated samples, compared to the controls. Two types of mitochondria were detected, often in the same cell. Organelles with darker matrix were usually narrow and elongated, whereas lighter ones were more rounded. In control cells, cristae were straight, narrow, and long in both types. Matrix granules were observable more frequent in darker than in lighter mitochondria (Figure 9Ca–c). Effect of rotenone treatment was different on the two types of mitochondria. In darker organelles, cristae swelled and formed swirls. Several wide crista junctions became identifiable. In lighter mitochondria, cristae disintegrated and often became unrecognizable (Figure 9Cd–i). Matrix granules disappeared or showed decreased density (Figure 9Cc,i).

Finally, we used an ER-stress assay to detect if the observed change is compound-specific or an “overall” cell death event is detected with the ATP or TUNEL assay. The XBP1 assay was performed to identify ER-stress in the ROT treated samples, where ER stress was not expected. As a positive control, tunicamycin was used. The XBP1 assays demonstrated that ROT (using EC50 concentration) has no ER-stress inducing effect in the D21, D28 or D42 neurospheres (Figure S6B), while cell death was observed in the cryosectioned ROT-treated samples (Figure 9A), providing a strong evidence that compound-specific effects can be determined in 3D spheroids upon treatment. Immunocytochemical investigation reflected some disorganization of the treated group of spheroids compared to the controls, however other marked differences were not observed (Figure S6A).

### 3.5. Neurite Outgrowth Assay is Suitable to Determine the Effect of NT Compounds in the 3D Model

**Figure 10:**
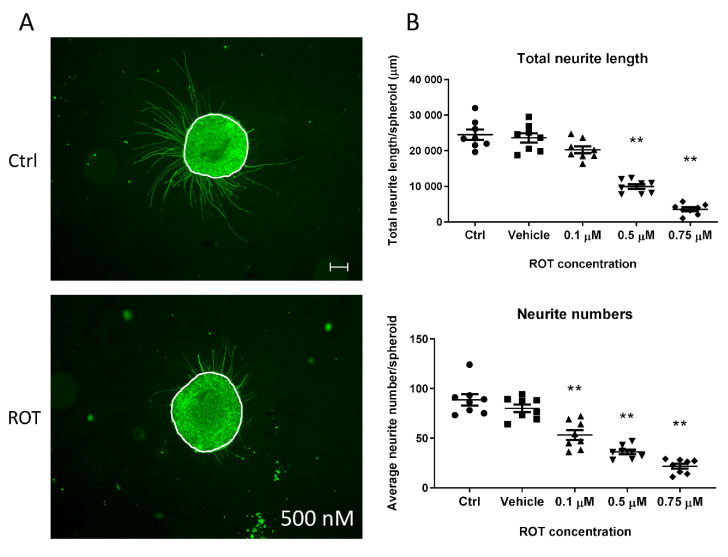
Neurite outgrowth measurement on D21 3D spheroids, exposed for 72 h with ROT. (**A**) Representative photograph of control (untreated) and ROT (0.5 µM) treated D21 spheroid immunolabeled with TUBB3 (in green). White lines represent the border of the spheroids where the neurite outgrowth was determined from, using ImageJ software (scale bar: 100 µm). (**B**) Total neurite length/spheroid (presented in µm ± SEM) and the average number of neurites/spheroids were determined 24 h after plating the treated spheroids. Different symbols denote treatment groups. (*n* = 3, in each experiment 8 spheroids were treated in each group) (** *p* < 0.01).

Functional readouts which connect the effect of a tested compound to a given cell type are essential to determine their tissue-specific effect. 3D spheroids represent a complex cell culture with a network of neurons, astrocytes, and oligodendrocytes, and this complexity makes more challenging to analyze minor differences in neurite length affected by chemical exposure due to the limitations of the current detection systems. Therefore, we investigated if neurite growth could be investigated using 3D neurospheres. We found that Matrigel-coated surface sufficiently supports the rapid and strong attachment of free-floating D21 3D neurospheres in order to generate a robust and reproducible procedure, where the effect of toxic compounds on neurite outgrowth can be investigated. The effect of different ROT concentrations were demonstrated comparing the total neurite outgrowth and the average number of neurites/spheroid ratio. ROT administration resulted significantly shorter and decreased number of neurites compared to the untreated and vehicle-treated control groups in all concentration levels in D21 spheroids (** *p* < 0.01) (Figure 10B).

## 4. Discussion

The development of in vitro platforms for neurotoxicology screenings is driven by the urgent needs of the chemical, food, cosmetic, and pharma industries. Most NT studies are carried out in rodents or rodent derived primary cells, resulting in relatively high cost and lower translational value of the results due to the species differences [3,46]. Major international initiatives have started to convert the traditional animal-based neurotoxicity tests to in vitro assays using both mammalian brain cells and human cells to detect and predict chemical hazards [47,48]. However, there is only a limited number of human neuronal cell lines (e.g., carcinoma cell lines such as SH-SY5Y; BE2-M17 or immortalized cell lines like LUHMES) are available and hard to obtain primary human CNS tissue suitable for NT studies. Overall, the highly complex structure of the human brain makes in vitro modeling very difficult. Human iPSCs could fill this niche and offer the advantage that other cells and tissue types (e.g., kidney, liver, cardiac, neuronal, intestinal) sharing the same individual genetic background can be created using specific differentiation protocols in a replicable manner. This may provide a very effective in vitro tool for toxicologist for capturing the individual variability in the human population [49]. For example, in 2D neurotoxicity screening, the usage of human iPSC derived neuronal cultures, especially the commercially available QC controlled neuron and astrocyte cultures, where the differentiation of iPSCs is not required for the “users”, is dynamically increasing [48].

In recent years, numerous in vitro models have been created in order to study the human CNS in a more physiological way, but the field of NT and DNT using 3D neuronal tissues did not develop as fast as disease or developmental modeling. It is due to the difficulty to find a compromise between biological complexity and technical reproducibility which are necessary for drug or toxicity screening [29].

An important approach was the application of human cell lines in NT assay development. For example, the LUHMES immortalized human fetal tissue-derived mesencephalic cell line can be efficiently differentiated towards dopamine-like neurons upon tetracycline administration [50,51] and provide a suitable system for neurotoxicology screenings [52,53] or Parkinson’s disease-related drug testing [54] both in 2D or 3D cultures [45,55,56,57]. Despite the advantages in straightforward handling, they represent only one specific cell type of the CNS. New developments using stem cell-derived astrocytes or microglia in co-culture with LUHMES cells could reveal new potential both in NT and DNT tests [58], but also highlight that full-PSC derived systems could provide great advantages over the conventional cell lines.

To develop an efficient, highly reproducible neurotoxicology test system, an improved 3D culture of microtissues differentiated from human PSCs would be beneficial. For example, in neuronal disease modeling, a major step that opened new perspectives, was the development of cortical layer-organized 3D brain microtissues, providing a complex system forming under in vitro conditions from human PSCs [59,60]. However, for toxicological studies, the very low throughput potential of such complex systems is a significantly limiting factor at the moment.

Huang and his colleagues demonstrated that human brain organoids could be applied as an in vitro model for CNS drug screening to evaluate structural, cellular, and molecular changes. They used neurotoxic tranylcypromine in hiPSC-derived brain organoids leading to decreased proliferation activity and apoptosis induction [61]. HiPSC-derived cerebral organoids were treated with different concentrations of vincristine for 48 h, and the expansion of the treated organoids was measured, showing concentration-dependent neurotoxicity. Vincristine inhibited fibronectin, tubulin, and MMP10 expression in the cerebral organoids, which was specific for its well-known effect on microtubule dynamics [62]. Recently, iPSC-derived cortical neurons and astrocytes were co-cultured in 3D to detect calcium oscillations upon a chemical compound treatment, analyzing multiple parameters, highlighting the potential of such readouts in neurotoxicity assessment [31]. Another study has presented a novel 3D heterotypic glioblastoma-brain sphere (gBS) model applied for screening new anti-glioblastoma agents [63]. This new application highlighted the flexibility of iPSC-derived platforms to be used in disease modeling, drug testing, and toxicology.

In this study, we have analyzed the complexity of our hiPSC-derived 3D neurosphere system demonstrating intensive glial-neuron interaction with the astrocytes and oligodendrocytes present in the neurospheres. Complex characterization was performed with gene expression, protein level analysis in line with immunocytochemical investigations, including morphological evidence of neurocrine communication at the end of the examined period. Morphologically, synapses are composed of precisely opposed pre- and postsynaptic membranes decorated with electron-dense thickening, synaptic cleft filled with a fine meshwork of electron-dense material, and presynaptic vesicles [64]. One of the two populations of presynaptic vesicles belong to the active zone matrix, is the docked vesicles [65]. The membrane of them is in contact with the presynaptic membrane, therefore they form the readily releasable pool (RRP) of synaptic vesicles. [66].

Our system showed similarities in morphology and maturation properties with a previous study where 3D organoids were generated and characterized over 8 weeks [29]. In our system, there was no need to use BDNF, GDNF, or a special electrophysiology media; a basal medium was sufficient to promote the differentiation of complex spheroids within 6 weeks of culture. Although the spheroids showed a continuous growth and development/maturation, the homogeneity of the plates was not compromised, both the diameter and total protein content of the spheroids within the 96-well plate showed very low variation at a given time point, represented by the low SEM values (Figure 1). Low variability of samples and high homogeneity is crucial when the aim is the development of a reliable HTS for DNT studies.

Here, we performed a medium-throughput 96-well plate assay on 3D neurospheres to detect the cytotoxic effect of the selected compounds, based on ATP-release measurements. We tested drugs, pesticides and well-known chemicals with or without neurotoxic effect in different stages of the neuronal differentiation. Our results highlighted a compound-specific and differentiation stage-related effect of the tested chemicals, providing the possibility to determine EC50 and EC10 values for the compounds (Table S3). For example, colchicine has a strong toxic effect on neurospheres with very similar EC50 values in all differentiation stages. Its effect can be detrimental, it blocks basic cellular (protein assembly, endocytosis, exocytosis, cellular motility, etc.) and neuronal functions at the same time (assembly of tubulin filaments in the microtubules of neurites), which we supposed to happen in all differentiation stages in our neurospheres. A comparable neurotoxic effect was described on iPSC derived 2D cultures of NSCs, neurons, and astrocytes [67], and LUHMES cells [57]. By comparing EC10 or EC50 values, another example is doxorubicin, where D42 samples were the most sensitive for the treatment when intensive protein synthesis could happen both in neurons and glial cells (e.g., neurotransmitter synthesis, axonal growth, oligodendrocyte maturation), therefore the potential blocking of the transcription machinery might have a major effect on cell viability (see Table 1 for a summary).

Continuing the line with hexachlorophene that blocks the electron transport chain, by acting on GLUD1, it affects the turnover of an excitatory neurotransmitter, glutamate. This effect could explain the observed differentiation stage-related sensitivity differences of the neurospheres, still under an acute exposure scheme. Likewise, less cytotoxic effect was reported on iPSC derived 2D cultures of NSCs, early neurons (14 days old) and astrocytes when 10 or 100 µM HE was exposed for 24 h [67], while more pronounced cytotoxicity and neurite outgrowth inhibition of LUHMES cells were reported [57].

Valproate is known as a DNT positive compound. However, in our cell viability assay, it has no detectable impact on the investigated concentration range, which might be in correlation with the maturity of the treated cells, they are not in early neurodevelopment (neural tube stage), but maturing neurons and astrocytes. When LUHMES cells were exposed the EC50 value of VPA was determined as >100 µM, and significant inhibition of neurite outgrowth was detected. While the EC50 value was in a similar range with our 3D neurosphere-driven data, we did not investigate its effect on neurite outgrowth. Here, we also have to clarify that cytotoxicity read-out alone is not suitable to predict DNT, although it is suitable to predict the neurotoxic effect of compounds or detect necrosis in differentiating cultures, more relevant end-points (e.g., proliferation, migration, apoptosis, network formation, synaptogenesis, and growth of neurites) must be investigated for evaluating the DNT effect of a compound [18].

In the case of pesticide rotenone, which inhibits mitochondrial Complex I of the electron transport chain, strong cell death was detected. The effect of ROT exposure was investigated extensively. In iPSC-derived neuron-astroglia 2D cell cultures the activation of the Nrf2/ARE pathway was documented upon ROT-induced oxidative stress, which led to the activation of astrocytes and cell death of neurons [67,78,79]. Using LUHMES cells this robust cytotoxic effect was not obvious while a prominent neurite growth inhibition was reported [57]. Using “BrainSpheres” system [30], where ICC-based morphology analysis, ROS measurements and viability were compared in different developmental times and exposure schemes, dopaminergic-neuron selective toxicity and general cytotoxic concentration were determined [30]. Importantly, increased cell death and mitochondrial dysfunction were detected in our ROT exposed 3D neurospheres, similar to others’ results [30]. In contrast, ibuprofen has no effect on neuronal cell viability in the investigated concentration range, as it was expected upon known in vitro and in vivo data.

Neurite outgrowth assays are well established in conventional 2D neuronal cultures, derived from cell lines, primary tissues, or iPSCs [57,80,81], however, the adaptation to 3D cultures is less tested. Determining the radial migration and neuronal density distribution within the migration area of NPCs grown as spheroids is rather described [27]. Here we adapted a simple system which provides a tissue-specific functional readout for neurospheres upon exposure, which can be automated using high content imaging (HCI) systems [45]. Based on our results, it is sensitive enough to distinguish different concentration of toxic compounds, does not require complicated read-out systems which was proved by testing the effect of ROT, previously confirmed to inhibit neurite growth of LUHMES cells [57]. Although these results agree with the reported neurite outgrowth inhibition effect, we cannot exclude the influence of cytotoxicity on the reduction of the overall neurite length we observed. Combining with LDH or lactate sampling, metabolite measurements from the media or terminal transcriptomic or proteomic assays, a complex dataset can be collected, and the effect of a given compound can be investigated systematically on the molecular level, as well to identify adverse effects. Similar to the RT-qPCR and ICC investigation that we used to characterize our 3D neurospheres these methods can be used when the effect of a given compound is investigated. Although we performed immunocytochemistry on treated spheroids using rotenone only, others successfully used such investigations to gain DNT readouts, for example detecting cell migration, neurite outgrowth, Ca^2+^ reabsorption, synaptogenesis, and PPAR pathway disruption [29,30,57,82,83].

Evaluating our concentration–response results, we faced with the lack of available in vitro human data, which makes the comparison challenging. This demonstrates that due to the lack of EC values and very different readouts, the appropriate comparison between the various test systems is often not possible. As summarized in Table 1, it is not possible to provide human in vitro NT values for every compound, the databases are incomplete. Although there are several initiatives and efforts on the field to collect and harmonize the available test methods and evaluate the data produced so far, there is still a lot to do in the field of in vitro neurotoxicity test method development [1,16].

## 5. Conclusions

We have established and evaluated a hiPSC-based 3D in vitro cellular model for the study of the neurotoxic effect of different compounds. Concentration–response was determined in different differentiation stages, using a well-known read-out the cell viability. For a certain compound, apoptotic activity, ER-stress assay and TEM was performed, providing possibilities to generate novel human CNS-relevant data for other compounds using hiPSC derived cells. Tissue-specific readouts such as neurite outgrowth were also investigated, however further, read-outs and DNT-relevant training compound set should be applied to evaluate the suitability of our 3D spheroids in DNT screening Nevertheless, due to the pluripotent nature of the hiPSC, this model offers an excellent tool for drug testing, gene therapy studies and toxicology studies parallelly on the same genotype using other cell or tissue types at the same time. Moreover, new advancement in gene manipulation such as CRISPR/Cas9 mediated gene targeting, makes it possible to target specific pathways and generate reporter cell lines for toxicological or other applications. Combining these new approaches with 3D cell culture-based assays could revolutionize the field of toxicology including DNT studies in the near future.

## Figures and Tables

**Table 1:** Known effects of the used compounds.

Compound Name (CAS Number)	Known Effects of the Compound
Acrylamide 79-06-1	- Water-soluble crystalline amide that polymerizes rapidly; - widely used in chemical industry (e.g., water treatment industry, paper industry, textile treatment industry) and cosmetics; - occurs in food upon heat treatment—both during home cooking or industrial processing of food— [68]; - neurotoxic: occupational exposures [69,70]; - could cross the placental barrier and appears in breast milk [71]; and - probable oral lethal human dose is between 50–500 mg/kg.
Colchicine 64-86-8	- Can bind to tubulin and inhibit tubulin polymerization leading to inhibition in mitosis; - interacts with the P-glycoprotein transporter (MDR1/ABCB1) and the CYP3A4 enzyme (both involved in toxin metabolism) [72,73]; - medication used to treat gout and Behçet’s disease; - probable oral lethal dose in humans is less than 5 mg/kg; and - in vitro cytotoxicity limit: 0.02 µM.
Doxorubicin 25316-40-9	- Intercalating chemotherapy drug; - inhibiting the movement of topoisomerase II, which leads to the blocking of both replication and transcription; and - LD50: 21.8 mg/kg (rat, subcutaneous).
Hexachlorophene 70-30-4	- Disinfectant; - blocks the electron transport chain through acting on membrane-anchoring subunit of succinate dehydrogenase (SDHD) and Glutamate dehydrogenase 1, mitochondrial (GLUD1); - probable oral lethal dose in humans is not determined; LD50: 66 mg/kg (rat, oral); and - in vitro cytotoxicity limit: 1.86 µM.
Ibuprofen 15687-27-1	- Drug (pain killer); - effectively reduces fever, as a non-steroid anti-inflammatory drug (NSAID), acts on inhibiting cyclooxygenase (COX) enzymes (COX-1 and 2); and - overdose symptoms appear in individuals consumed more than 99 mg/kg; LD50: 636 mg/kg (rat, oral).
Mercury(II) chloride 7487-94-7	- Component of pesticides; - corrosive, toxic; - could accumulate in the kidney; - probable oral lethal dose is 5–50 mg/kg; and - in vitro cytotoxicity limit: 1.37 µM.
Paracetamol 103-90-2	- Drug (pain killer); - ioverdosing could cause liver toxicity; and - hepatic toxicity in humans occurred with acute overdoses more than 10 g; LD50: 2400 mg/kg (rat, oral).
Paraquat 7 5365-73-0	- Herbicide; - neurotoxic: occupational exposures leading to Parkinson’s disease [74]; - widely investigated neurotoxic mechanism [75,76]; and - probable oral lethal dose in humans is 35 mg/kg.
Rifampicin 13292-46-1	- Antibiotic; - stops RNA synthesis in bacteria; - could cause liver toxicity; - robable lethal oral dose in humans is 14–60 gLD50: 1570 mg/kg (rat); and - in vitro cytotoxicity limit: 4.37 µM.
Rotenone 83-79-4	- Pesticide; - inhibits mitochondrial Complex I of the electron transport chain; - in rats Parkinson’s disease- like symptoms were developed, →considered as an environmental risk factor for PD [77]; - probable lethal dose in human 0.3–0.5 g/kg; and - in vitro cytotoxicity limit: 0.22 µM.
Valproic acid 1069-66-5	- Drug used in epilepsy, bipolar disorders, and for the prevention of seizures; - known to block voltage-gated sodium channels and increase brain levels of gamma-aminobutyric acid (GABA); - well-known teratogen induces different congenital malformations and neural tube defects (NTDs); and - LD50: 670 mg/kg (rat, oral).

Note: probable lethal dose data is provided upon PubChem database (https://pubchem.ncbi.nlm.nih.gov/), in vitro multicellular cytotoxicity “comptox” data were collected from EPA’s Chemical dashboard (https://comptox.epa.gov/dashboard).

## Data Availability

Additional files are made available online along with the manuscript.

## Supplementary Materials

The following are available online at https://www.mdpi.com/2073-4409/9/5/1122/s1, Figure S1: Characterization of CTRL-2 hiPSC line, Figure S2: Characterization of NPCs, Figure S3: Summary of the morphological differentiation of neurospheres, Figure S4: Representative immunostaining of cryosectioned neurospheres, Figure S5: The initial delta Ct distributions of relevant neuronal markers in D2 stage samples by Real-time PCR measurements, Figure S6: Effect of ROT treatment on 3D spheroids, Table S1: Antibodies used for immunocytochemistry and flow cytometry, Table S2: Primers used in the study, Table S3: Tested compounds and their concentration, Table S4: Effective concentration (EC) values of compounds tested on D21, D28 and D42 3D neurospheres.

## Abbreviations

3D	three dimensional;
AO	adverse outcome;
BSA	bovine serum albumin;
CAS	chemical abstracts service;
CNS	central nervous system;
Ctrl	control;
D	day;
DNT	developmental neurotoxicity;
EC	effective concentration;
ER	endoplasmic reticulum;
FBS	fetal bovine serum;
HE	Hematoxylin-Eosin;
HTS	high-throughput screening;
hiPSC	human induced pluripotent stem cell;
ICC	immunocytochemistry;
iPSC	induced pluripotent stem cell;
NA	numeric aperture;
NAM	new approach method;
NEAA	non-essential amino acids;
NIM	neural induction media;
NMM	neural maintenance media;
NPC	neural progenitor cell;
NSC	neural stem cell;
O/N	overnight;
PBMC	peripheral blood mononuclear cells;
PBS	phosphate buffer saline;
PCR	polymerase chain reaction;
PFA	Paraformaldehyde;
POL/L	Poly-l-ornithine and Laminin;
PSC	pluripotent stem cell;
RT	room temperature;
rTdT	recombinant terminal deoxynucleotidyl transferase;
RT-PCR	reverse transcription polymerase chain reaction;
TEM	transmission electron microscopy;
TUNEL	terminal deoxynucleotidyl transferase dUTP nick end labeling.
