# Supplementary material for: Human Induced Pluripotent Stem Cell-Derived 3D-Neurospheres Are Suitable for Neurotoxicity Screening
Source: Cells. 2020 May 1;9(5):1122. doi: 10.3390/cells9051122 (PMC7290365; doi:10.3390/cells9051122)
Supplement: Supplementary file 1 [file cells-09-01122-s001.zip › Kobolak et al_Supplementary Figures_revised.pdf]

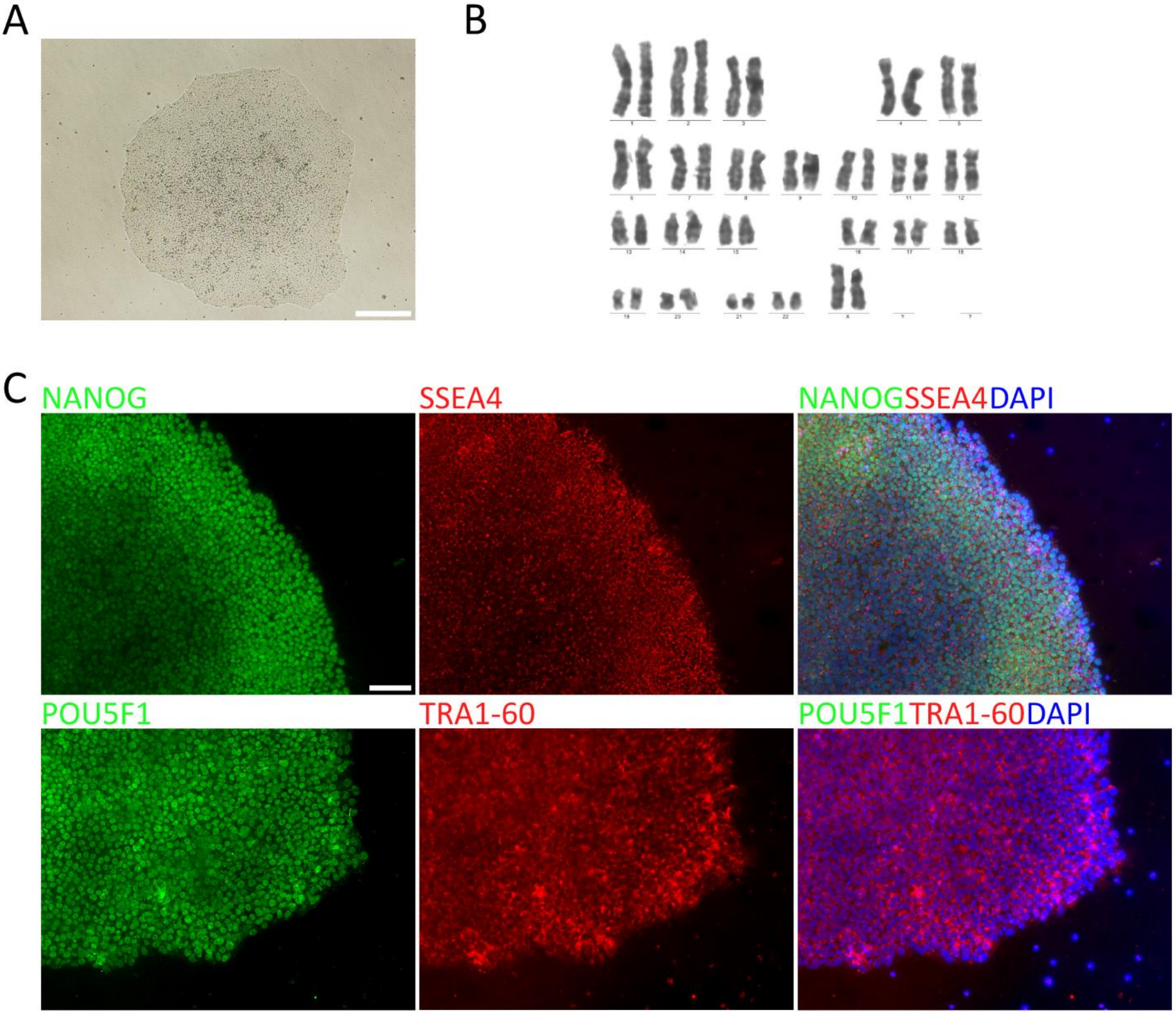

Figure S1 Characterisation of CTRL-2 hiPSC line. A) CTRL-2 hiPSC colony grown on Matrigel coated surface in mTeSR1 media. Scale bar: 100µm) **B**) Normal dipoid karyotype (46XX) detected in the CTRL-2 cell line during karyotype analysis. **C**) Representative immunostaining of iPSCs labelled for NANOG (green) SSEA4 (red) POU5F1 (green) and Tra1-60 (red). Nuclei were counterstained with DAPI. Scale bar: 100µm.

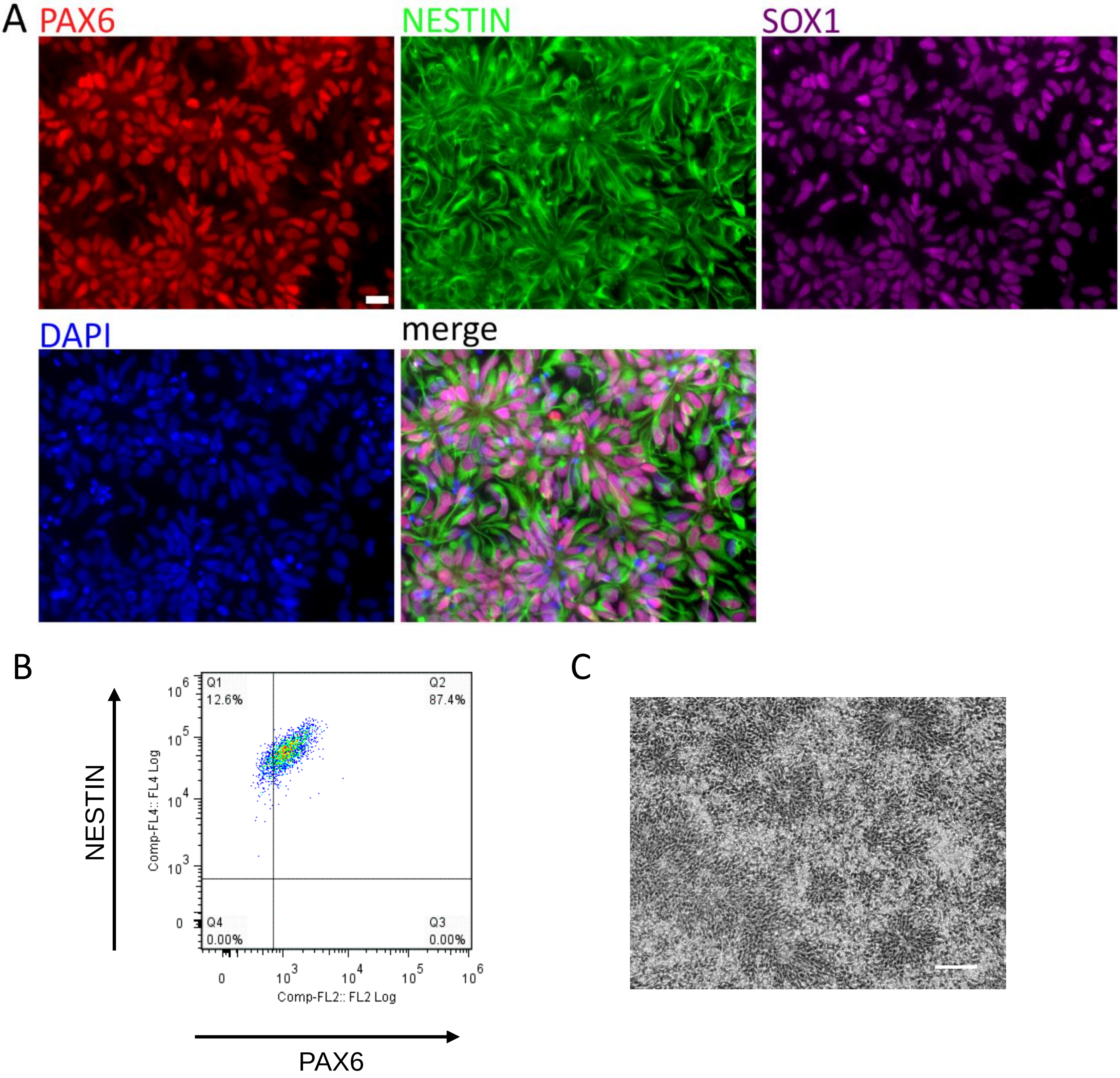

Figure S2 Characterisation of NPCs. **A**) Representative immunostaining of NPCs (p4) labelled for PAX6 (red) NESTIN (green) and SOX1 (magenta). Nuclei were counterstained with DAPI. Merge image represents the colocalization of the labelled proteins. Scale bar: 20µm **B**) Flow cytometry analysis of NPCs labelled with PAX6 and NESTIN as described in Zhou et al. 2016 (PMID: 27321088) **C**) Phase contrast image of confluent NPC culture (p4). Scale bar: 100µm

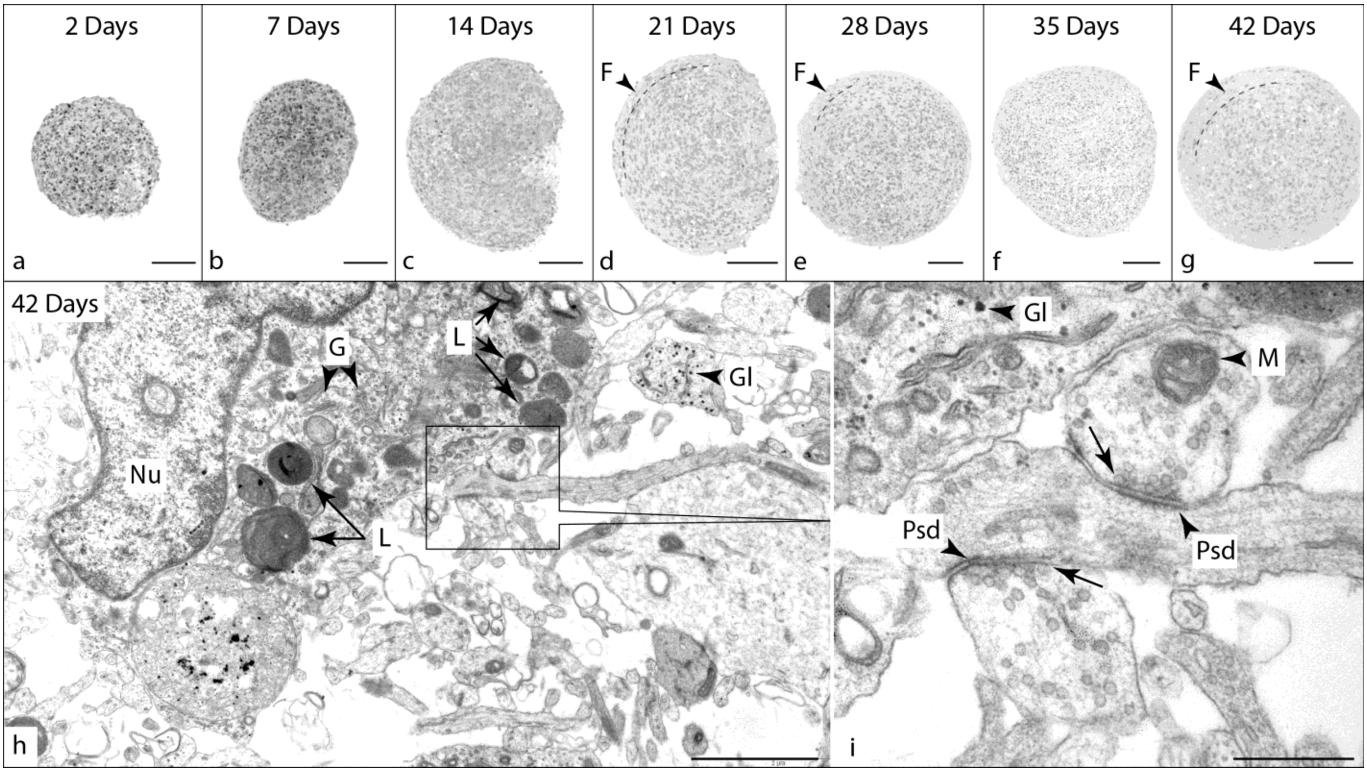

Figure S3 Summary of the morphological differentiation of neurospheres. a-g) Overview of neurospheres (semithin sections at the maximum diameter of cultures). a-c) Initially, cell density is high and consistent. d-g) Lighter layer that mainly consists of fibres (F) appears on the surface of developing cultures. h) A cell and its environment in the D42 culture. i) Magnified part of the h panel with two synapses. Presynaptic axon terminals, pre- and postsynaptic membranes, and presynaptic vesicles are clearly visible. Note the docked vesicles indicating active zone (black arrows) (G – Golgi stacks, Gl – glycogen granules, L – late lysosomes, M – mitochondrion, Nu – nucleus, Psd – postsynaptic density; scale bars: a-g: 100µm, h: 2µm, i: 500nm)

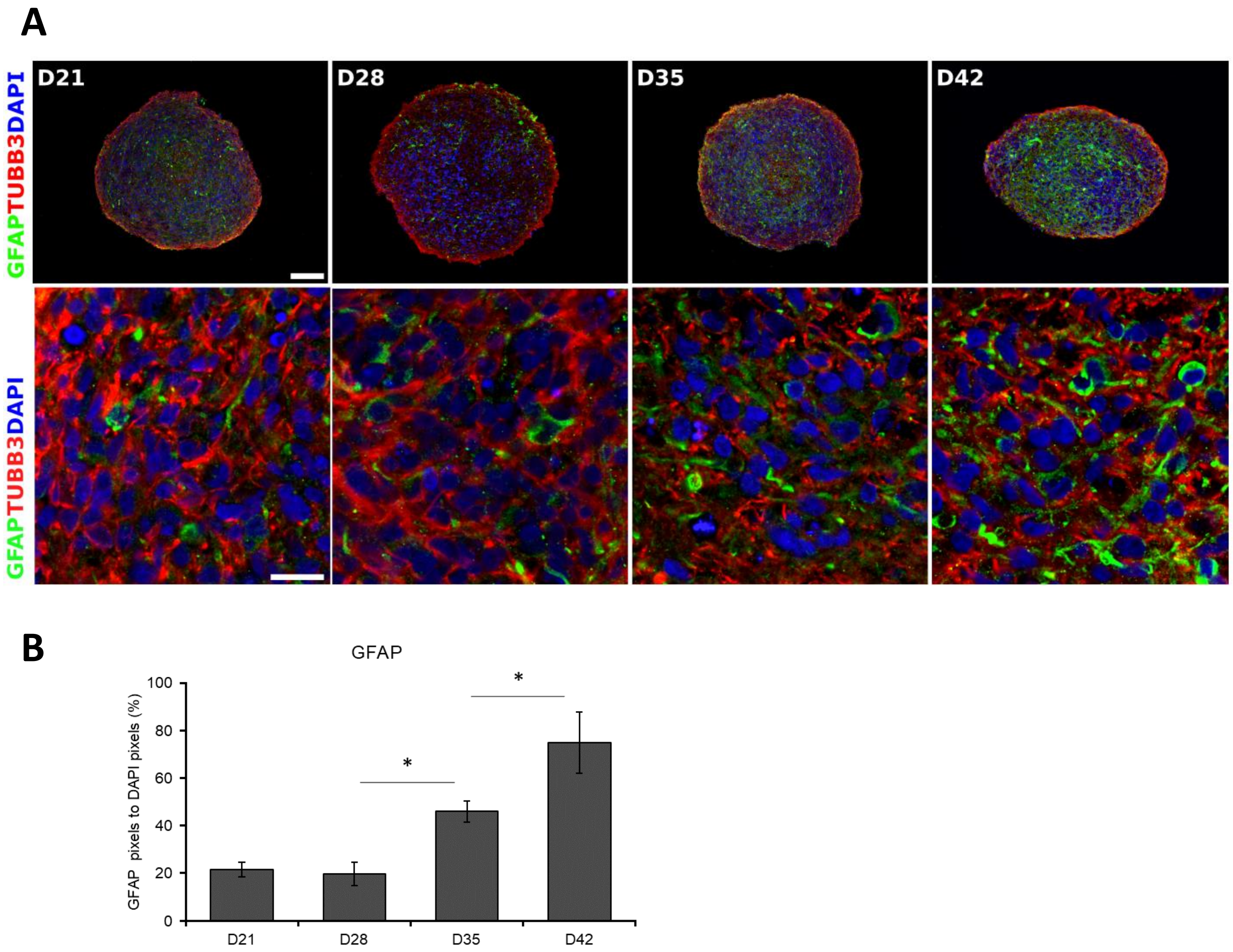

Figure S4 Representative immunostaining of cryosectioned neurospheres A) labelled for GFAP (green) and TUBB3 (red) (first and second line) presenting the astrocytes in the 3D neurospheres at D21, D28, D35 and D42 stage. Nuclei were counterstained with DAPI. Scale bar: 100µm and 25µm. B) Quantification of GFAP confocal images were performed, the immunoreactive pixels were measured in 5 neurospheres (middle sections, 5 randomly selected fields/slide) at every time points. Data was normalized with DAPI positive nuclei number. Data were expressed as percentage of marker/DAPI ratio ±SEM (*$p<0.05$).

Gene	ΔCt	Std error
NES	1.3	0.26
PAX6	6.1	0.03
SOX1	3.2	0.06
SOX9	6.1	0.12
TUBB3	2.3	0.07
MKI67	4.3	0.22
MAP2	1.8	0.03
MAPT	8.8	0.03
NEUN	14.9	0.00
DLG4	6.8	0.23
GFAP	15.8	0.07
CLDN11	12.6	0.25

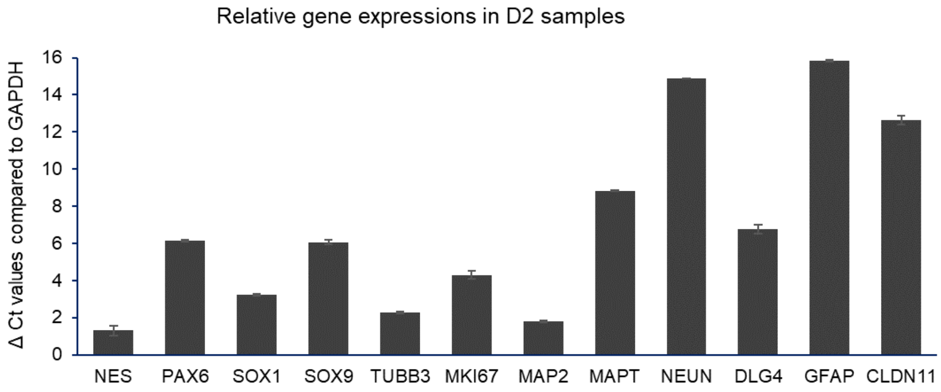

Figure S5 The initial delta Ct distributions of relevant neuronal markers in D2 stage samples by Real-time PCR measurements. Values were determined by Livak's Delta Ct method for visualization the neuronal marker expressions in the phase of D2, compared to GAPDH as an internal control gene (Livak and Schmittgen, 2001; PMID: 11846609). Mean values ±SD are presented on graphs.

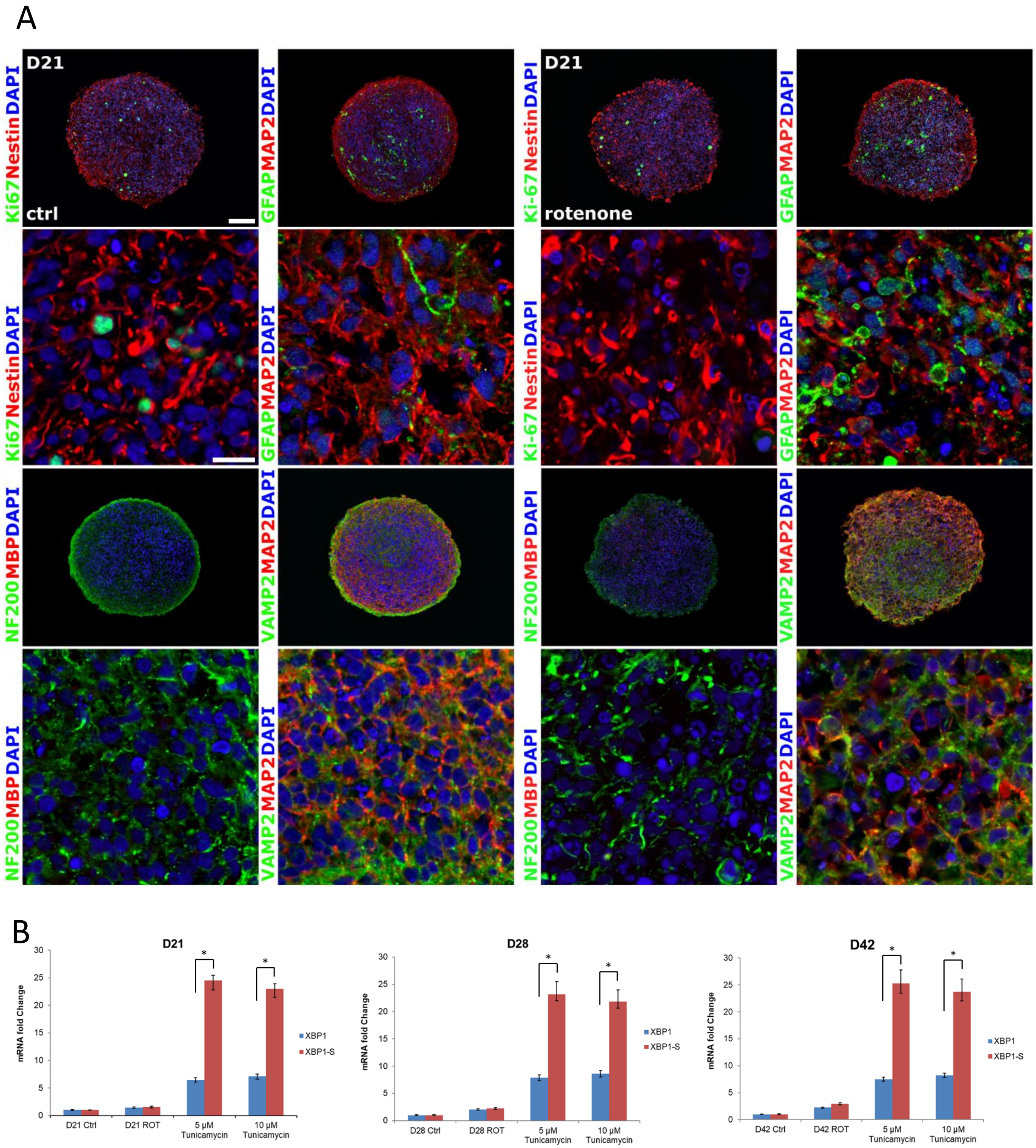

Figure S6 Effect of ROT treatment on 3D spheroids. A) Representative immunostaining of cryosectioned Ctrl (vehicle (0.1% DMSO) treated) and 0.5µM ROT treated D21 neurospheres. Cryosections were labelled with Ki67/NESTIN, APQ4/MAP2, NF200/MBP and VAMP2/MAP2. Protein name IDs are indicated with colours, representing the colour of the used fluorophore (e.g. green as Alexa 488; red as Alexa 594). Nuclei were counterstained with DAPI (in blue). Scale bar: 100µm and 25µm **B**) ER-stress was investigated in samples to prove the specific effect of ROT. As expected, no ER-stress was detectable in the treated samples. As a positive control Tunicamycin treatment was performed, using two different concentrations. Average values ±SEM are presented on the graphs (*p< 0.05).
