# Supplementary material for: Human Induced Pluripotent Stem Cell-Derived 3D-Neurospheres Are Suitable for Neurotoxicity Screening
Source: Cells. 2020 May 1;9(5):1122. doi: 10.3390/cells9051122 (PMC7290365; doi:10.3390/cells9051122)
Supplement: Supplementary file 1 [file cells-09-01122-s001.zip › Kobolak et al_Supplementary_Tables.pdf]

Supplementary Tables

Table S1. Antibodies used for immunocytochemistry and flow cytometry

	Antibody	Antibody Registry* Identifier	Dilution	Company (Cat #; LOT #)
**Neuronal markers**	Mouse anti-NESTIN	AB_2251134	1:1000	Merck (MAB5326; 2919678)
	Rabbit anti-PAX6	AB_291612	1:250	Covance (PRB278P; B201255)
	Rabbit anti-MAP2	AB_94856	1:1000	Merck (MAB3418; 2654330)
	Mouse anti-TUBB3	AB_1119489	1:1000	Santa Cruz (sc58888; B1815)
	Rabbit anti-NF200-KD	AB_306298	1:1000	Abcam (ab8135; GR3183142-8)
	Rabbit anti-VAMP2	AB_2798240	1:250	Cell Signalling (13508; 01/2018)
	Rabbit anti-Vglut1/2	AB_2285905	1:500	Synaptic Systems (135503; 135503/8)
	Rabbit anti-GAD65/67	AB_880149	1:1000	Abcam (ab49832; GR135533-2)
	Rabbit anti-VAChT	AB_887864	1:500	Synaptic Systems (139103; 2-46)
	Rabbit anti-TH	AB_297840	1:500	Abcam (ab112; 920883)
	Rabbit anti-AQP4	AB_2274338	1:100	Santa Cruz (sc20812; E3014)
	Rabbit anti-GFAP	AB_305808	1:500	Abcam (ab7260; GR3202608-2)
	Mouse anti-MBP	AB_325006	1:20	AbD Serotec (MCA686S; 260505)
**Others**	Rabbit anti-Ki-67	AB_302459	1:200	Abcam (ab16667; GR216200-2)
**Secondary Antibodies**	Alexa Fluor 488 donkey anti-mouse IgG	AB_141633	1:600	Thermo Fisher (A21202; 1890861)
	Alexa Fluor 488 goat anti-rabbit IgG	AB_143165	1:600	Thermo Fisher (A11008; 1869581)
	Alexa Fluor 594 donkey anti-mouse IgG	AB_2535789	1:2000	Thermo Fisher (A21203; 1820027)
	Alexa Fluor 594 donkey anti-rabbit IgG	AB_141637	1:3000	Thermo Fisher (A21207; 1890862)

* http://antibodyregistry.org/

Table S2. Primers used in the study

Gene Symbol	Gene ID		Primer Sequence (5'-3')	PCR product (bp)
**RT-qPCR primers**				
PAX6	5080	F:<br>R:	GCCAGCAACACACCTAGTCA<br>TGTGAGGGCTGTGTCTGTTC	138
NES	10763	F:<br>R:	ACTGAAGTCTGCGGGACAAG<br>CAGTGGTGCTTGAGTTTCTG	64
SOX1	6656	F:<br>R:	TAGTAAGGCAGGTCCAAGCA<br>GGGTGGTGGTGGTAATCTCT	98
SOX9	6662	F:<br>R:	GTGCTCAAAGGCTACGACTG<br>TTGACGTGCGGCTTGTTC	80
TUBB3	10381	F:<br>R:	AACGAGGCCTCTTCTCACAA<br>GGCCTGAAGAGATGTCCAAA	107
MAP2	4133	F:<br>R:	TTGTCTCTAACCGAGGAAGCA<br>TCGTTGTGTCGTGTTCTCAA	103
MKI67	4288	F:<br>R:	ACGGATTATACCTGGCCTTC<br>AGGAAGCTGGATACGGATGT	180
MAPT	4137	F:<br>R:	TTGGAACTGCTGCCATGATT<br>GGCACAAGTCCTTACAAAGAGAAC	82
DLG4	1742	F:<br>R:	ACAAGCGGATCACAGAGGAG<br>AGTCTCTCTCGGGCTGGAAC	187
RBFOX3	146713	F:<br>R:	GGAGAAGCTGAATGGGACGA<br>GCCGTGGCATTATTGACCT	60
AQP4	361	F:<br>R:	ATGGAGGTGGAGGACAACAG<br>GGTCAACGTCAATCACATGC	85
CLDN11	5010	F:<br>R:	TTCCTGAAATCCTCAATTCATC<br>ACATCATACAAACCTGAAATCAAA	133
GRIN1	2902	F:<br>R:	GCAACACCAACATCTGGAAG<br>ATCCGCATACTTGGAAGACA	68
CHAT	1103	F:<br>R:	GCCTGCTGCAATCAGTTCTT<br>GTCCTCGTTGGAAGCCATT	114
TH	7054	F:<br>R:	TCCACGCTGTACTGGTTCAC<br>GCACCATAGGCCTTCACCTC	71
GAD1	2571	F:<br>R:	GCACAGGTCATCCTCGATTT<br>TTGATGTCAGCCATTCTCCA	81
SLC6A4	6532	F:<br>R:	GTGAGGATGTGGATGGAGGT<br>GCGAGATAGCATCCCTGTTC	191
GAPDH[1]	2597	F:<br>R:	CTCTCTGCTCCTCCTGTTCGAC<br>TGAGCGATGTGGCTCGGCT	69
**RT-qPCR and RT-PCR primers used for XBP1-assay**				
XBP1(S)[2]	7494<br>(NM_001079539.1)	F:<br>R:	TGCTGAGTCCGCAGCAGGTG<br>GCTGGCAGGCTCTGGGGAAG	169
XBP1(S) /<br>*XBP1*(U)	7494<br>(NM_001079539.1;<br>NM_005080.3)	F:<br>R:	GGGAATGAAGTGAGGCCAGT<br>GGAAGGGCATTTGAAGAACA	183/209

[1] The reference gene used in this study. [2] van Schadewijk et al. 2012; F, Forward; R, Reverse

Table S3. Tested compounds and their concentration

Compound name [chemical name]	CAS number	Supplier (Cat.N.)	Purity	Stock conc. (mM)	Stock solvent	Tested conc. (µM)
Paraquat dichloride hydrate [1,1′-Dimethyl-4,4′-bipyridinium dichloride hydrate]	75365-73-0	Carbosynth (FP40644)	≥ 98%	100	water	1000, 600, 300, 100, 30, 10, 1
Doxorubicin hydrochloride	25316-40-9	Sigma-Aldrich (D1515)	≥ 98.0%	200	water	200, 100, 30, 10, 1, 0.1, 0.01
Paracetamol [4′-Hydroxy-acetanilide]	103-90-2	Sigma-Aldrich (A7085)	≥ 99%	100	DMSO	100, 50, 30, 10, 1, 0.1, 0.01
Rotenone	83-79-4	TCI Chemicals (R0090)	96.5 %	100	DMSO	100, 30, 10, 1, 0.1, 0.01, 0.001
Ibuprofen [α-Methyl-4-(isobutyl)phenylacetic acid]	15687-27-1	TCI Chemicals (I0415)	99.9%	100	DMSO	100, 30, 10, 1, 0.1, 0.3, 0.01
Valproic acid [2-Propyl-pentanoic acid]	1069-66-5	Sigma-Aldrich (P4543)	≥ 98%	400	water	400, 200, 100, 50, 30, 10, 1
Acrylamide [2-Propen-amide]	79-06-1	Sigma-Aldrich (A3553)	≥ 99%	3500	water	3500, 2000, 1000, 600, 300, 100, 10
Rifampicin [3-(4-Methyl-piperazinylimino-methyl)rifamycin]	13292-46-1	Carbosynth (AR11351)	≥ 96.5%	100	DMSO	100, 30, 10, 1, 0.1, 0.03, 0.01
Mercury(II) chloride	7487-94-7	Carbosynth (FM35310)	≥ 99.5%	1000	DMSO	1000, 600, 100, 30, 10, 1, 0.1
Hexachlorophene [2,2′-Methylenebis(3,4,6-trichlorophenol)]	70-30-4	TCI Chemicals (M0219)	99.5%	100	DMSO	100, 10, 1, 0.3, 0.1, 0.03, 0.01
Colchicine [(S)-N-(5,6,7,9-Tetrahydro-1,2,3,10-tetramethoxy-9-oxobenzo[a]heptalen-7-yl)acetamide]	64-86-8	Carbosynth (FC11732)	≥ 94%	50	water	10, 1, 0.1, 0.01, 0.001, 0.0003, 0.0001

Table S4. Effective concentration (EC) values of compounds tested on D21, D28 and D42 3D neurospheres

Compounds	D21 (log µM)			D28 (log µM)			D42 (log µM)		
	**EC50** ±SD	**EC25** ±SD	**EC10** ±SD	**EC50** ±SD	**EC25** ±SD	**EC10** ±SD	**EC50** ±SD	**EC25** ±SD	**EC10** ±SD
Colchicine (COL)	-0.49 ±0.17	-2.04 ±0.21	-3.59 ±0.37	-0.64 ±0.11	-1.89 ±0.15	-3.16 ±0.24	-0.92 ±0.14	-2.24 ±0.18	-3.55 ±0.29
Rotenone (ROT)	-0.61 ±0.08	-1.25 ±0.10	-1.89 ±0.16	-0.74 ±0.08	-1.46 ±0.12	-2.19 ±0.18	-0.62 ±0.13	-1.48 ±0.18	-2.35 ±0.27
Doxorubicin (DOX)	0.67 ±0.07	0.04 ±0.10	-0.58 ±0.15	1.11 ±0.06	0.72 ±0.11	0.33 ±0.17	1.32 ±0.07	0.9 ±0.11	0.49 ±0.18
Hexachlorophene (HE)	1.26 ±0.08	0.53 ±0.10	-0.2 ±0.16	1.01 ±0.05	0.21 ±0.06	-0.59 ±0.09	0.5 ±0.09	-0.34 ±0.10	-1.18 ±0.16
Paraquat (PQ)	1.89 ±0.07	1.52 ±0.10	1.15 ±0.15	1.98 ±0.05	1.67 ±0.08	1.35 ±0.12	1.79 ±0.08	1.44 ±0.11	1.09 ±0.18
Mercury(II) chloride (HgCl2)	1.87 ±0.05	1.5 ±0.06	1.1 ±0.10	1.83 ±0.03	1.60 ±0.05	1.4 ±0.07	1.74 ±0.03	1.54 ±0.04	1.34 ±0.05
Paracetamol (PAR)	>2	>2	>2	>2	>2	>2	>2	>2	>2
Ibuprofen (IBU)	>2	>2	>2	>2	>2	>2	>2	>2	>2
Rifampicin (RIF)	>2	>2	>2	>2	>2	>2	>2	>2	>2
Valproic acid (VPA)	>2.6	>2.6	>2.6	>2.6	>2.6	>2.6	>2.6	>2.6	>2.6
Acrylamide (ACR)	>3.5	>3.5	>3.5	>3.5	>3.5	>3.5	>3.5	>3.5	>3.5
